# Nitric oxide regulates cardiac intracellular Na^+^ and Ca^2 +^ by modulating Na/K ATPase via PKCε and phospholemman-dependent mechanism^☆^

**DOI:** 10.1016/j.yjmcc.2013.04.013

**Published:** 2013-08

**Authors:** Davor Pavlovic, Andrew R. Hall, Erika J. Kennington, Karen Aughton, Andrii Boguslavskyi, William Fuller, Sanda Despa, Donald M. Bers, Michael J. Shattock

**Affiliations:** a Cardiovascular Division, King's College London, The Rayne Institute, St. Thomas' Hospital, London, UK; b Division of Cardiovascular and Diabetes Medicine, Medical Research Institute, College of Medicine Dentistry and Nursing, University of Dundee, UK; c Department of Pharmacology, University of California Davis, CA, USA

**Keywords:** NO, nitric oxide, PKC, protein kinase C, PLM, phospholemman, VASP, vasodilatory protein, VF, ventricular fibrillation, l-NAME, N(G)-nitro-l-arginine methyl ester, ARVM, adult rat ventricular myocytes, EGTA, ethylene glycol tetraacetic acid, BDM, 2,3-butanedione monoxime, Bis, bisindolylmaleimide, PLB, phospholamban, GC, guanylate cyclase, Nitric oxide, Protein kinase C, Phospholemman, FXYD-1, Sodium pump, Arrhythmia

## Abstract

In the heart, Na/K-ATPase regulates intracellular Na^+^ and Ca^2 +^ (via NCX), thereby preventing Na^+^ and Ca^2 +^ overload and arrhythmias. Here, we test the hypothesis that nitric oxide (NO) regulates cardiac intracellular Na^+^ and Ca^2 +^ and investigate mechanisms and physiological consequences involved. Effects of both exogenous NO (via NO-donors) and endogenously synthesized NO (via field-stimulation of ventricular myocytes) were assessed in this study. Field stimulation of rat ventricular myocytes significantly increased endogenous NO (18 ± 2 μM), PKCε activation (82 ± 12%), phospholemman phosphorylation (at Ser-63 and Ser-68) and Na/K-ATPase activity (measured by DAF-FM dye, western-blotting and biochemical assay, respectively; *p* < 0.05, *n* = 6) and all were abolished by Ca^2 +^-chelation (EGTA 10 mM) or NOS inhibition l-NAME (1 mM). Exogenously added NO (spermine-NONO-ate) stimulated Na/K-ATPase (EC50 = 3.8 μM; *n* = 6/grp), via decrease in *K*_m_, in PLM^WT^ but not PLM^KO^ or PLM^3SA^ myocytes (where phospholemman cannot be phosphorylated) as measured by whole-cell perforated-patch clamp. Field-stimulation with l-NAME or PKC-inhibitor (2 μM Bis) resulted in elevated intracellular Na^+^ (22 ± 1.5 and 24 ± 2 respectively, vs. 14 ± 0.6 mM in controls) in SBFI-AM-loaded rat myocytes. Arrhythmia incidence was significantly increased in rat hearts paced in the presence of l-NAME (and this was reversed by l-arginine), as well as in PLM^3SA^ mouse hearts but not PLM^WT^ and PLM^KO^. We provide physiological and biochemical evidence for a novel regulatory pathway whereby NO activates Na/K-ATPase via phospholemman phosphorylation and thereby limits Na^+^ and Ca^2 +^ overload and arrhythmias. This article is part of a Special Issue entitled “Na^+^ Regulation in Cardiac Myocytes”.

## Highlights

• We tested whether nitric oxide regulates intracellular Na^+^ and Ca^2 +^ in the heart.
• Nitric oxide increased Na/K ATPase activity via PKCε-induced phospholemman phosphorylation.
• Inhibiting nitric oxide pathway resulted in Na^+^ and Ca^2 +^ overload and contributed to arrhythmia development in the heart.

## 1 Introduction

Maintenance of diastolic intracellular [Na^+^] ([Na]*_i_*) and [Ca^2 +^] ([Ca]*_i_*) is crucial for the normal cardiac function. In the heart, [Na]*_i_* indirectly controls [Ca]*_i_* via Na/Ca exchanger, thus, regulating contractility. With each action potential, Na^+^ enters the cell through voltage gated Na^+^ channels and the only quantitatively significant mechanism for extruding this Na^+^ is the Na/K-ATPase. Na/K-ATPase stimulation reduces [Na]*_i_* and thus indirectly [Ca]*_i_* (via Na/Ca exchanger), allowing the cell to maintain diastolic [Ca]*_i_* in the range of 100–200 nM. Regulation of the cardiac Na/K-ATPase occurs via the FXYD accessory protein phospholemman (PLM)[1–3]. Unphosphorylated PLM tonically inhibits Na/K-ATPase and this inhibition is relieved by phosphorylation at Ser-63, Ser-68 or Ser/Thr-69 by PKC [4,5] or at Ser-68 by PKA [1,2,6]. PKA activation results in Na/K-ATPase stimulation via Ser-68 PLM phosphorylation, thus, limiting [Na]*_i_* and [Ca]*_i_* and reducing the propensity for triggered arrhythmias [7] during fight or flight. The physiological role of PKC-induced Na/K-ATPase stimulation has not been established. Raising intracellular Ca^2 +^ either artificially or through pacing-induced contraction activates constitutively expressed nitric oxide synthase (NOS), generating nitric oxide (NO) in submicromolar concentrations [8,9]. Confusingly, NO has been reported to mediate both inhibition [10–15] and stimulation of the Na/K-ATPase [16–21]. In the present study, we have examined the effects of NO on the Na/K-ATPase activity, [Na]*_i_* and [Ca]*_i_* in ventricular myocytes and whole hearts. We have also investigated the signaling pathway involved in the modulation of Na/K-ATPase by NO.

## 2 Methods

Detailed methodology is provided in the SI for techniques such as single ventricular myocyte contractility, Ca^2 +^ transients, intracellular Na^+^ measurements [7], endogenous NO synthesis, western blotting [5], Na/K-ATPase assay [1,22], electrophysiology and PKC activation. Adult rat ventricular myocytes (ARVM) were isolated from the hearts of adult male rats and PLM^WT^, PLM^3SA^ (Genoway, France) and PLM^KO^, PLM^WT^ (University of Virginia Transgenic Facility) mice by standard collagenase enzymatic digestion [22,23]. PLM^3SA^ mouse is a novel knock-in mouse line globally expressing an unphosphorylatable form of PLM in which residues 63, 68 and 69 have been mutated to alanines (see SI for details). Myocytes were field-stimulated for up to 20 min at 20 V and 2 or 3 Hz, in the presence of a number of pharmacological agents. Rat hearts were paced at 300 bpm or 600 bpm and mouse hearts at 550 and 800 bpm (5 ms pulse, 1.5 times threshold) via a unipolar electrode inserted into the base of the left ventricle with reference to the metal aortic cannula and were monitored for arrhythmias using heart rate variability (HRV) software (HRV module, ADI instruments, US). HRV software scores the extent of arrhythmias from 0 to 25 (arbitrary units) and ventricular fibrillation is scored as 30. In separate experiments in rat hearts, VF threshold was determined using a protocol adapted from Zaugg et al. [24]. Quantitative data are shown as mean ± standard error of the mean (SEM). Differences between experimental groups were tested by one-way ANOVA followed by a Bonferroni post-hoc test or by paired or unpaired *T*-tests. In arrhythmia studies, VF incidence was compared using Fisher's exact test and contingency tables. In experiments measuring VF threshold, pilot experiments showed that within heart variability was normally distributed (as assessed by the Kolmogorov-Smirnov test for normality); however, as previously shown by others [24], variation in VF threshold between hearts was logarithmically distributed. Log_10_ VF thresholds were therefore compared by one-way ANOVA followed by a post-hoc Student–Newman–Keuls test. Differences were considered significant at *p* < 0.05. It should be noted that while the rat heart clearly can sustain VF, the ability of the mouse to do so may be limited by the small size of the heart and the perfusion conditions [25]. We have therefore used a different method to assess arrhythmias in the mouse hearts. Heart rate variability (HRV) analysis software was used to measure the variability of the inter-beat interval. Standard deviation of change in adjacent beat-to-beat intervals (i.e. SD of ΔNN) was derived and this was used to give the value reported as an “Arrhythmia Score.”

## 3 Results

### 3.1 Endogenous NO induces PLM and PLB phosphorylation

**Fig. 1:**
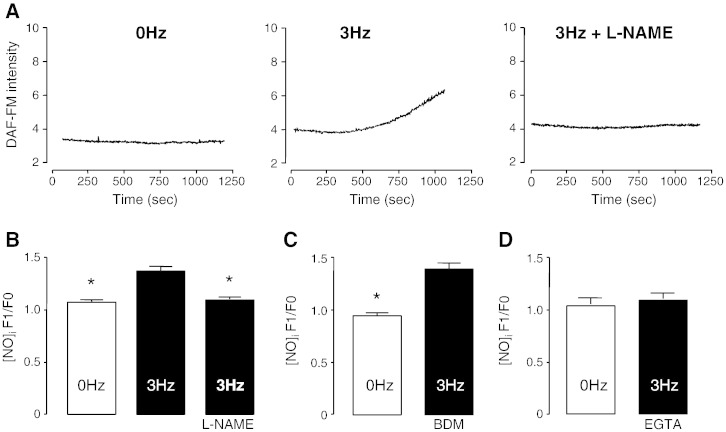
Nitric oxide is synthesized during field-stimulation. DAF-FM fluorescence raw traces following field-stimulation (3 Hz) of rat ventricular myocytes (A). Graph of DAF-FM fluorescence changes in the presence of 1 mmol/L l-NAME (B), 2.5 mmol/L BDM (C) and 10 mmol/L EGTA (D). The data represent cells isolated from at least 6 individual animals and are expressed as mean ± SEM (*P < 0.05).

In order to determine whether field-stimulation results in endogenous NO synthesis, ARVM were loaded with NO sensitive fluorescent dye and paced from rest for 20 min at 3 Hz. As expected, field-stimulation induced a significant increase in [NO]*_i_* and this increase was abolished by the general NOS-inhibitor, l-NAME (1 mmol/L), as shown in Figs. 1A and B (*n* = 6/grp). Increase in [NO]*_i_* was not due to movement artifacts as it could not be abolished in the presence of 2.5 mmol/L of myofilament-desensitizer BDM (Fig. 1C). However, removal of extracellular Ca^2 +^ with 10 mmol/L EGTA completely abolished NO synthesis (Fig. 1D). We infer that it is Ca^2 +^ and not action potential or contraction that mediates the rise in NO.

**Fig. 2:**
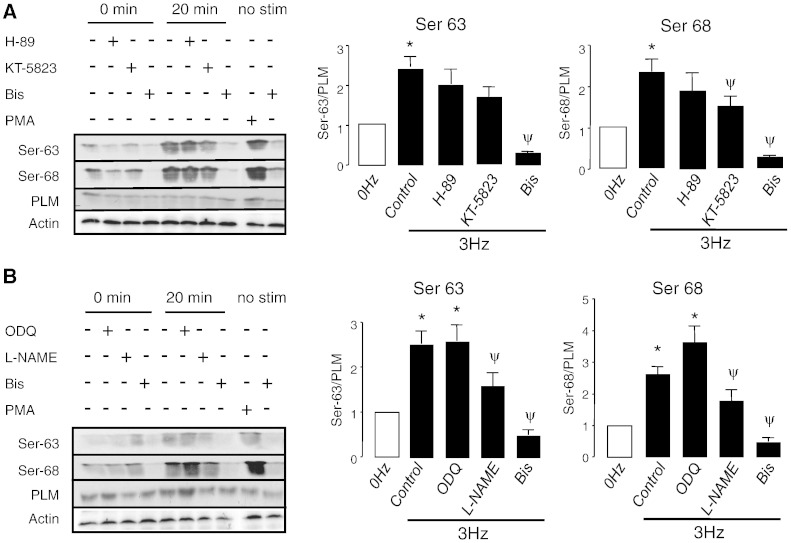
PLM phosphorylation via NOS/PKC activation. Western blots showing changes in PLM expression and phosphorylation, following field-stimulation (at 3 Hz, 20 min) of rat ventricular myocytes in the presence of 2 μmol/L H-89, 1 μmol/L KT-5823 or 2 μmol/L Bis (A) and 1 mmol/L l-NAME, 1 μmol/L ODQ or 2 μmol/L Bis (B). The data are normalized to total expression, represent cells isolated from at least 6 individual animals and are expressed as mean ± SEM (*P < 0.05 compared to 0 Hz; ^y^*P* < 0.05 compared to 20 min pacing control).

Interestingly, field-stimulation (3 Hz) resulted in sustained increases in PLM phosphorylation (Na/K-ATPase regulatory protein) at Ser-63 and Ser-68 (Fig. 2A and B) but no change was observed in total PLM or Ser-69 residue (see Fig. S1A). It should be noted that in rat, PLM 69 residue is a threonine and in a mouse, 69 is serine. However, since this residue is neither strongly basally phosphorylated [26] nor does it respond to pacing, this is unlikely to confound our studies. Similarly, increases were observed in phosphorylation of SERCA2a regulatory protein phospholamban (PLB) at Ser-16 and Thr-17 residues (Fig. S1B). PKA agonist (forskolin) was used as a positive control and resulted in increase in PLB Ser-16 phosphorylation, but not Thr-17 (Fig. S1B, left panel). Field-stimulation-induced phosphorylation of PLB at Thr-17 [27,28] and Ser-16 [29] was observed by others.

### 3.2 PLM phosphorylation occurs via Ca^2 +^/NO-dependent PKCε activation

To investigate the signaling pathways mediating PLM phosphorylation, pharmacological agents were assessed for their ability to reduce PLM phosphorylation at Ser-63 and Ser-68, following 20 min of pacing (Fig. 2A). Whereas PKA-inhibitor H-89 (2 μmol/L) and PKG-inhibitor KT-5832 (1 μmol/L) had no significant effect on Ser-63 phosphorylation, PKC inhibitor Bis (2 μmol/L) completely abolished pacing-induced PLM phosphorylation at both Ser-63 and Ser-68. PKC-agonist PMA (300 nmol/L) was used as a positive control (Fig. 2A, left panel, last two lanes). No change in PLM phosphorylation was observed in the presence of CaMKII-inhibitor KN-93 (2 μmol/L) as shown in Fig. S2. KN-93 reduced PLB phosphorylation at Thr-17 residue, a known CaMKII substrate (Fig. S2A).

As NO can signal through a cGMP/sGC/PKG [30] we examined whether PLM phosphorylation is sensitive to NOS and sGC inhibitors, using l-NAME (1 mmol/L) and ODQ (1 μmol/L), respectively (Fig. 2B). l-NAME significantly reduced phosphorylation at both Ser-63 and Ser-68 (Fig. 2B, middle and right panel). However, ODQ had no effect on PLM phosphorylation, suggesting that sGC and PKG are not required.

**Fig. 3:**
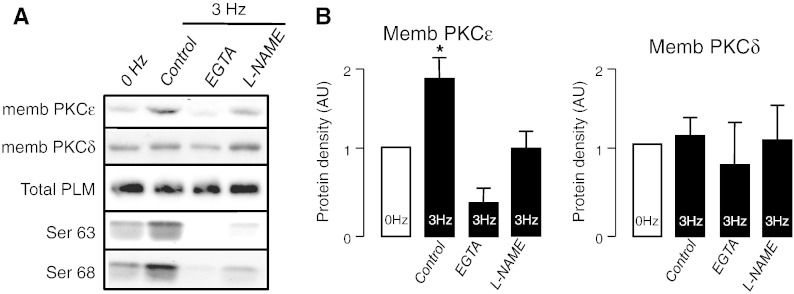
NO activates PKCε-isoform. Western blots showing PKCε and PKCδ translocation following 20 min of field-stimulation (A). Changes in membranous fraction of PKCε and PKCδ following 20 min of field-stimulation (B). The data represent cells isolated from 5 individual animals and are expressed as mean ± SEM (*P < 0.05 compared to 0 Hz control).

We tested whether endogenous NO can induce PKC activation by examining the extent of translocation of PKCε and PKCδ from cytosolic to membranous fraction as a result of field stimulation. PKCε membrane fraction increased, whereas no significant translocation of PKCδ was observed (Figs. 3A,B). PKCε translocation (following 20 min of field-stimulation) was abolished by either chelating extracellular Ca^2 +^ (using EGTA) or by NOS inhibition (using l-NAME), whereas no change was observed in PKCδ (Figs. 3A,B). Similar to our previous data, pacing-induced PLM phosphorylation at Ser-63 and Ser-68 was abolished in the presence of EGTA and l-NAME, whereas total PLM expression was unaltered (Fig. 3A). PKCε but not PKCδ membrane fraction increased (and cytosolic fraction decreased) in a time dependent manner when myocytes were paced from quiescence, whereas, PMA (300 nmol/L) treatment of non-stimulated cells, alone, induced a significant translocation of both PKCε and PKCδ (Fig. S3).

### 3.3 NO stimulates Na/K-ATPase in a PLM-dependent manner

**Fig. 4:**
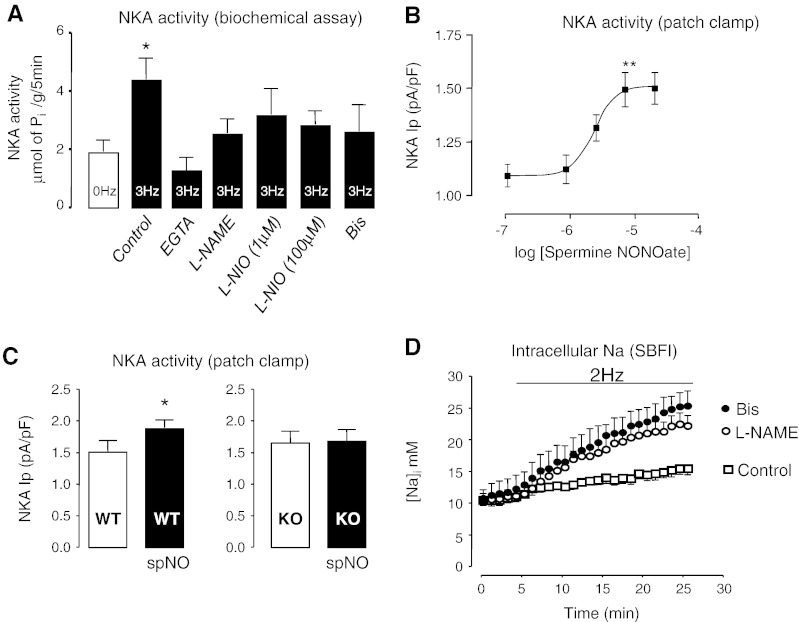
NO increases Na/K-ATPase activity and reduces Na^+^ overload. Na/K-ATPase activity in adult rat cardiac myocytes field-stimulated for 20 min in the presence of 10 mmol/L EGTA, 1 mmol/L l-NAME, 1 and 100 μmol/L l-NIO or 2 μmol/L Bis (A). Effects of spermine NONO-ate on Na/K-ATPase *I*_p_ in rat myocytes, using patch clamp (B). Effects of 10 μmol/L spermine NONO-ate on Na/K-ATPase *I*_p_ in mouse PLM^WT^ and PLM^KO^ myocytes, using patch clamp (C). [Na]*_i_* in paced rat cardiac myocytes in the presence of 1 mmol/L l-NAME or 2 μmol/L Bis, using SBFI (D). The data represent cells isolated from at least 6 individual animals and are expressed as mean ± SEM (*P < 0.05 compared to control).

To test whether endogenous NO increases Na/K-ATPase activity, Na/K-ATPase activity was measured in field-stimulated (3 Hz) ARVM using a biochemical Na/K-ATPase assay. Na/K-ATPase activity was significantly higher in paced vs. non-paced cells (4.40 ± 0.73 vs. 1.89 ± 0.40 μmol/mg/5 min; *n* = 7), as shown in Fig. 4A. This increase was abolished in the presence of EGTA (10 mmol/L), l-NAME (1 mmol/L) and Bis (2 μM), indicating that pacing-induced increase in Na/K-ATPase activity is due to Ca^2 +^/NOS-dependent PKC activation. In order to dissect contributions of eNOS and nNOS isoforms, Na/K-ATPase activity was measured at 1 and 100 μM of l-NIO. Both concentrations resulted in Na/K-ATPase inhibition (Fig. 4A), consistent with eNOS but not excluding nNOS involvement.

To test whether exogenous NO can activate Na/K-ATPase activity in quiescent rat and mouse myocytes, Na/K-ATPase pump current (*I*_p_) was measured in the presence of an NO donor (spermine-NONO-ate), using whole-cell perforated patch-clamp technique. Patch pipette [Na^+^] was either 30 or 100 mmol/L, allowing detection of changes in apparent Na^+^ affinity or *V*_max_. Spermine-NONO-ate significantly increased *I*_p_ in ARVM at 10 and 30 μmol/L in the presence of 30 mmol/L of patch pipette (Fig. 4B). Furthermore, 10 μmol/L of spermine NONO-ate significantly increased *I*_p_ in PLM^WT^ but not PLM^KO^ (Fig. 4C) indicating that NO-induced increase in *I*_p_ is PLM-dependent. The stimulatory effect of spermine NONO-ate was lost when pipette Na^+^ was increased to 100 mmol/L (see Fig. S4A) suggesting that the effects of NO on the Na/K ATPase activity are mediated by a decrease in *K*_m_ for Na^+^ rather than the increase in *V*_max_. 10 μmol/L of “spent” spermine-NONO-ate (depleted of NO) had no effect on the *I*_p_, showing that increases in *I*_p_ were not due to spermine artifacts (see Fig. S4B). Furthermore, no effect of NO on *I*_p_ was observed when PLM^3SA^ myocytes were treated with 10 μmol/L of spermine NONO-ate (Fig. S4C) showing that the NO-induced stimulatory effect requires PLM phosphorylatable residues.

### 3.4 NO regulates intracellular Na^+^ and Ca^2 +^

In order to analyze whether NO affects [Na]*_i_* and [Ca]*_i_*, ARVM were field-stimulated in the presence of NOS or PKC inhibitors. It should be noted that due to the toxicity of the SBFI dye to the paced cardiac myocytes over prolonged field-stimulation periods, 2 Hz field-stimulation frequency was used for these experiments. There was a significant increase in [Na]*_i_* in l-NAME or Bis treated cells, compared to non-treated controls (Fig. 4D). Thus, NO-dependent Na/K ATPase activation pathway acts to limit the rise in [Na]*_i_* in a beating ARVM. Increases in [Na]*_i_* can induce increases in [Ca]*_i_* via Na/Ca exchanger and thus contribute to arrhythmogenesis [7]. Therefore, not surprisingly, in field-stimulated ARVM l-NAME resulted in sustained increases in Ca^2 +^ transients (100 ± 21 %) and sarcomere length shortening (circa 81 ± 19.4 %), compared to non-treated cells (Figs. S5A and 5B), as well as spontaneous Ca^2 +^ transients occurring between triggered beats (Fig. S5C).

**Fig. 5:**
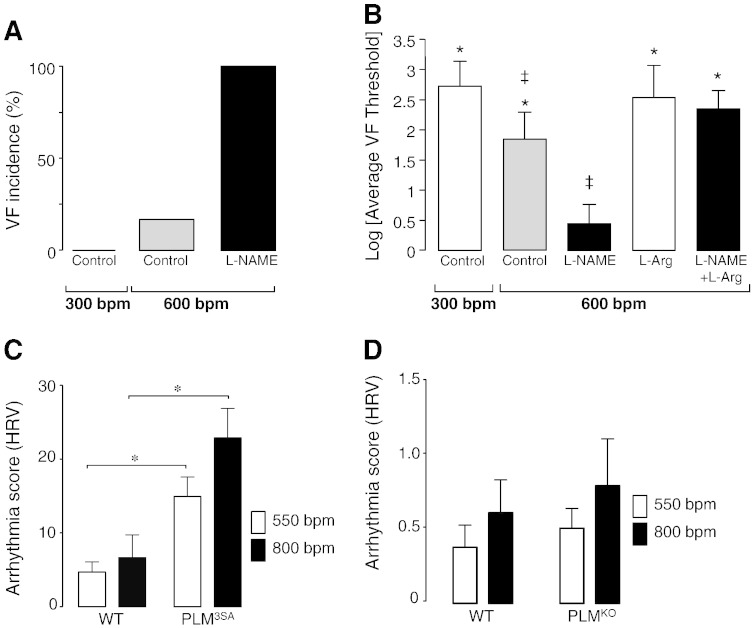
Incidence of spontaneous arrhythmias during pacing in whole hearts. Aerobically perfused rat hearts were subjected to 15 min of rapid pacing at 600 bpm (control at ~ 300 bpm) in the presence or absence of 300 μmol/L l-NAME (A). Data are shown as % of total number of hearts that spontaneously went into VF (*n* = 6), and were compared by Fisher's exact test and contingency tables. *P* < 0.05 as considered significant. VF threshold in paced rat hearts perfused with control or test solutions (B). VF threshold was determined three times over 15 min of rapid pacing in each heart (*n* = 8). The average VF threshold for each individual heart was calculated, and data are shown as the mean ± SEM of the log of these values. Data were compared by a one-way ANOVA test, and individual comparisons were conducted by a Student–Newman–Keuls post-hoc test. (*P < 0.05 compared to l-NAME treated group; ^‡^*P* < 0.05 compared to 300 bpm group). Arrhythmia incidence in paced mouse WT and PLM^3SA^ hearts (C). Arrhythmia incidence in paced mouse WT and PLM^KO^ hearts (D). Aerobically perfused mouse hearts were subjected to 10 min of rapid pacing at 550 bpm followed by 10 min of rapid pacing at 800 bpm and assessed for arrhythmias using HRV software. Data are shown as arrhythmia scores and were compared by one-way ANOVA test. *P* < 0.05 as considered significant.

In order to assess the physiological significance of this NO pathway at physiological heart rates and its ability to protect against arrhythmias, isolated rat hearts were paced at 300 and 600 bpm in the presence or absence of l-NAME (300 μmol/L). Pacing at 600 bpm caused 1 out of 6 hearts to spontaneously fibrillate, however, in the presence of l-NAME 6 out of 6 hearts developed ventricular fibrillation (VF) as shown in Fig. 5A. Indeed, VF threshold data demonstrate that VF threshold is significantly reduced with l-NAME and this reduction could be abolished by outcompeting l-NAME with l-arginine (Fig. 5B).

In order to directly test whether NO-induced PLM phosphorylation is at least in part responsible for this anti-arrhythmic effect, we have generated a transgenic mouse where PLM phosphorylation residues were mutated to alanines (PLM^3SA^). In PLM^3SA^ mice, Na/K pump activity cannot be increased via kinase-mediated PLM phosphorylation. Indeed, addition of PKA agonist forskolin had no effect on the *I*_p_ in PLM^3SA^ mice but increased *I*_p_ in PLM^WT^ mice (see Figs. S6A and 6B). It should be noted that PLM^3SA^ mice showed a significant decrease in PLM expression and a small but non-significant increase in Na/K ATPase α-1 and α-2 isoform expression (Figs. S6C and S6D). Thus, through decrease in PLM/Na/K-ATPase ratio, PLM^3SA^ mice have maintained unchanged basal Na/K ATPase activity under non-stimulated conditions. Mouse PLM^WT^ and PLM^3SA^ hearts were paced at 550 and 800 bpm and the incidence of arrhythmias was quantified using heart rate variability software. PLM^3SA^ hearts paced at both 550 and 800 bpm showed significantly higher susceptibility to arrhythmias, compared to their wild-type littermates (Fig. 5C). Not surprisingly, there were no significant differences in arrhythmia scores between PLM^KO^ and their wild-type littermates (Fig. 5D), considering that PLM^KO^ hearts differ from the PLM^3SA^ hearts in that they have a disinhibited sodium pump [2] and are thus protected against Na^+^ and Ca^2 +^ overload.

## 4 Discussion

**Fig. 6:**
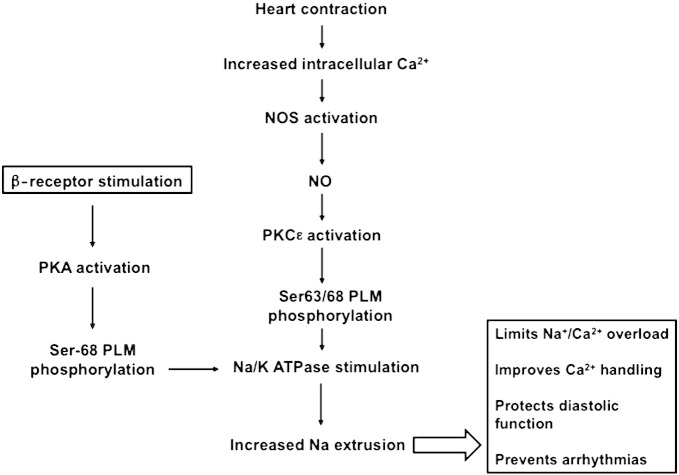
Schematic diagram of the proposed signaling pathway.

The role of NO signaling has been well defined in blood vessel dilation and neuronal transmission, however despite many observed effects; the integrated role of NO in regulating cardiac function is ambiguous. The plethora of effects includes the modulation of cardiac contractility but, confusingly, NO has been reported to have both negative [31,32] and positive inotropic effects [11,33,34]. The specific nature of the subcellular mechanisms underlying these diverse effects in the heart is largely unknown or contradictory. The present study provides evidence that endogenous NO helps maintain Na^+^ and Ca^2 +^ homeostasis and thus prevent arrhythmias in both field-stimulated isolated myocytes and field-stimulated whole hearts at physiological frequencies. We show that NO activates Na/K-ATPase via PKCε-induced phosphorylation of PLM at Ser-63 and Ser-68. We also show that exogenously added NO increases apparent Na^+^ affinity of Na/K-ATPase in rat myocytes in a dose-dependent manner. The effect on *I*_p_ observed in the presence of an NO donor was found to be PLM-dependent. NO in the isolated heart was shown to be protective and inhibition of the NO pathway either via NO inhibition or PLM mutation was found to be pro-arrhythmic. Taken together, these results suggest a novel endogenous pathway (Fig. 6), whereby endogenous NO stimulates the Na/K-ATPase via PLM phosphorylation, and thus protects the heart against Na^+^ and Ca^2 +^ overload and arrhythmias at physiological heart rates.

### 4.1 Pacing-induced stimulation of NO synthesis

Previously, it has been shown that raising intracellular Ca^2 +^ either artificially or through field-stimulation activates constitutively expressed NOS, generating NO in submicromolar concentrations [8,9]. In the present study, pacing induced an increase in [NO]*_i_* with increases in DAF-FM fluorescence corresponding to those observed in the presence of 40 μM exogenously applied spermine-NONO-ate (Fig. S8C). l-NAME, a general NOS inhibitor, abrogates the pacing-induced increase in [NO]*_i_*, indicating that NO is synthesized through NOS activation. In turn, this NOS activation is dependent on Ca^2 +^, as NO synthesis is blocked by chelation of extracellular Ca^2 +^ with 10 mmol/L EGTA. In the presence of EGTA, both Ca^2 +^ influx and Ca^2 +^-induced-Ca^2 +^-release (via ryanodine receptors) are prevented while the action potential, albeit attenuated, is likely to be maintained suggesting it is Ca^2 +^, rather than depolarization that is required for NOS activation. Importantly, in myocytes where Ca^2 +^ was still present but sarcomere shortening was mechanically uncoupled using BDM, increases in [NO]*_i_* were still observed (Fig. 1C) indicating that the increase in [NO]*_i_* is Ca^2 +^-dependent and not due to contraction or dye-concentration artifacts. It should be noted that at this concentration BDM does not completely abolish sarcomere shortening but rather reduces it by approximately 95%. Higher concentrations of BDM were not used due to its ability to deplete Ca^2 +^ from the SR. Nevertheless, no inhibition or indeed reduction in NO synthesis was observed compared to the field-stimulated controls, suggesting that Ca cycling was not affected.

### 4.2 Endogenous NO stimulates Na/K-ATPase via PLM phosphorylation

NO has been reported to both inhibit and stimulate Na/K-ATPase. It is likely that differences in the NO donors used and the concentrations of NO generated may add to the ambiguous nature of NO. Indeed, Vila-Petroff and colleagues have shown that NO donor, S-nitroso-N-acetylpenicillamine, produces biphasic contractile effects in cardiac tissue, with a positive inotropy at low NO concentrations and negative inotropy at high concentrations [32]. In this study, inhibition of endogenous NO resulted in positive inotropy. The effects of NO investigated here are likely to be more physiologically relevant as endogenous NO synthesis pathways are activated by field-stimulation. It is possible that inherent NO instability can result in the formation of peroxynitrite, which has been shown to directly inhibit Na/K-ATPase [35,36]. In our experiments NO stimulates Na/K-ATPase by increasing its apparent Na^+^ affinity, which is in agreement with the study by William et al. [16]. However, whereas William and colleagues suggest that NO-induced Na/K-ATPase stimulation is PLM-independent, we observed that this stimulation occurs *via* PKCε-induced PLM phosphorylation at residues Ser-63 and Ser-68. Indeed, we observed no stimulation in NO-treated PLM^KO^ (Fig. S4C) and PLM^3SA^ animals (Fig. 4C).

When applied to field-stimulated ARVM, l-NAME caused a dramatic reduction in endogenous NO synthesis (Fig. 1B), PKCε activation (Fig. 3D), PLM phosphorylation (Figs. 2A and B) and Na/K-ATPase activity (Fig. 4A). Specifically, l-NAME caused an inhibition of Na/K-ATPase activity by 43% whereas l-NIO, a more selective eNOS inhibitor (at a concentration of 1 μM has an 8-fold selectivity for eNOS [37]) reduced Na/K-ATPase activity by 29%. One hundred micromolar of l-NIO (at 100 μM, l-NIO inhibits both eNOS and nNOS) further reduced Na/K-ATPase activity (compared to the inhibition observed with 1 μM of l-NIO) indicating a possible involvement of both eNOS and nNOS isoforms. Similarly, in rapidly paced cat ventricular myocytes, NO was produced as a result of both eNOS and nNOS activation [38]. However, due to the inherent non-selective nature of NOS inhibitors, these data should be confirmed in eNOS and nNOS mutants. It should be noted that Na/K-ATPase activity assay is a blunt tool and involves the assessment of ouabain-sensitive ATPase activity on a very significant non-specific background ATPase activity in cardiac myocytes. This gives this assay an inherent variability (*i*.*e*. large error bars) which makes resolving differences at the lower end of the range more difficult, as demonstrated by its inability to resolve reduction of Na/K ATPase activity below baseline (non-paced) using Bis as shown in Fig. 4A.

### 4.3 PKCε as a terminal kinase

The signaling pathway through which NO exerts its effects is a subject of debate. In many systems, NO has been shown to signal through (i) a cGMP-dependent pathway (for review, see Fischmeister et al.)[30], (ii) cGMP-independent pathway, (iii) *via* S-nitrosylation (for review, see Stamler et al.)[39] or (iv) *via* an increase in cAMP through activation of PKA [32,40]. Here we show that endogenously synthesized NO increases PLM phosphorylation at residues Ser-63 and Ser-68 via PKCε activation. Although, PKG inhibitor KT-5823 caused a small reduction in PLM phosphorylation at Ser-68, sGC inhibitor (ODQ) did not reduce PLM phosphorylation suggesting that PKG is not the critical terminal kinase. Similarly, William et al. found that NO-induced increase in Na/K-ATPase *I*_p_ was not sensitive to ODQ [16]. Furthermore, we have previously shown that recombinant PKG1α is not able to phosphorylate PLM *in vitro* [41]; however, other PKG isoforms are yet to be tested. It is likely that the PKG inhibitor KT-5832 at a concentration used in this study partially inhibits PKC. The *K_i_* of KT5823 is 234 nmol/L for PKG and 4 μmol/L for PKC; thus, some PKC inhibition at the concentration used here (1 μmol/L) is expected and this is likely to account for the slight reduction in PLM phosphorylation observed here. Indeed, KT5832 at a lower concentration of 600 nmol/L had no effect on PLM phosphorylation (Fig. S2). KT5832 did, however, reduce phosphorylation of a known PKG substrate vasodilator-stimulated phosphoprotein (VASP) at Ser-239 residue [42] as shown in Fig. S2. Similarly, more specific PKG inhibitor, RP-8-bromo-cGMPs (100 μmol/L), reduced VASP Ser-239 phosphorylation but had no effect on PLM phosphorylation (Fig. S2). Thus, whilst contraction of cardiac myocytes activates both PKC and PKG pathways via NO, only PKCε kinase appears to be involved in the phosphorylation of PLM at residues Ser-63 and Ser-68. In field-stimulated cells, [Ca]*_i_* will increase and this rise in [Ca]*_i_* can enhance both NOS and PKC activity (for review, see Lammerding et al. 2004)[43]. Indeed, we show that Ca^2 +^ chelation with EGTA prevents NO production (Fig. 1D).

Ping and colleagues found that NO (either endogenous NO produced during ischemia or exogenous NO generated by NO donors) induces late phase ischemic preconditioning by activating PKCε, via S-nitrosylation [44,45]. Indeed, our data demonstrate that field-stimulation induces activation of PKCε but not PKCδ and this PKC translocation is dependent on Ca^2 +^ and NOS activation as shown in Fig. 3. Therefore, it is reasonable to conclude that the terminal kinase involved in pacing-induced PLM phosphorylation is PKCε rather than PKG. Furthermore, we have previously demonstrated that receptor-mediated PKC activation in adult rat ventricular myocytes elicits a similar PLM phosphorylation profile to the one observed in this study, with sustained Ser-63 and Ser-68 phosphorylation [5].

### 4.4 Functional consequence of pacing-induced NO mediated PLM phosphorylation

Results in Figs. 5A and B demonstrate that pacing of rat hearts (at physiological heart rates) has little effect on VF threshold *per se*; however, when NOS is inhibited, pacing is profoundly pro-arrhythmic with 100% of hearts going into VF. This suggests that endogenous NO exerts a strong protective effect against arrhythmias. NO may exert a range of other protective effects including PLB phosphorylation [34,46] (and the associated improvement in SR Ca handling), altered L-type Ca channel function [47,48], reduced RyR leak [49] and improved mitochondrial metabolism [38]. However, the mechanism described in this study (*i*.*e*. the phosphorylation of PLM and resulting stimulation of Na/K-ATPase) clearly contributes to the anti-arrhythmic effect of NO as shown by data in Fig. 5C. We provide direct evidence that inactivation of this NO-dependent Na^+^/Ca^2 +^ regulatory pathway (via PLM^3SA^ mutation) results in elevated diastolic intracellular Ca^2 +^ levels, compared to their WT littermates (Fig. S7A). Importantly, in PLM^3SA^ mouse myocytes, field-stimulation in the presence and absence of β-adrenergic receptor stimulation resulted in significantly higher susceptibility to arrhythmias, compared to their WT littermates (Fig. S7B). Fig. S7B shows examples of spontaneous Ca^2 +^ transients occurring between triggered beats in myocytes from PLM^3SA^ mice. Such spontaneous Ca^2 +^ transients occurred in 4/19 cells in the absence, and 17/19 cells in the presence of β-adrenergic receptor stimulation, indicating that both NO and PKA-mediated regulatory pathways are required for maintenance of Na^+^ and thus Ca^2 +^ homeostasis (see Fig. 6 for proposed pathway). Whether this mechanism is present *in vivo* where sympathetic stimulation may dominate, and if so, to what extent, remains to be investigated. At submaximal levels of β-receptor agonist (isoprenaline), effects of endogenous NO and sympathetic stimulation on PLM phosphorylation are additive (see Fig. S8). These data show that PKA and PKC pathways can act in concert and have an additive effect on PLM phosphorylation at residue Ser-68, providing evidence that two pathways are not conflicting as previously reported for other substrates with cAMP and cGMP [30]. While the contribution of each pathway to the maintenance of intracellular Na^+^ has not been determined, this may depend on the prevailing conditions. It is also possible that Na^+^ accumulation causes a mild acidification and that this could contribute to arrhythmias seen under these conditions. To what extent this occurs remains to be investigated.

The most likely substrates (other than PLM) that could contribute to the anti-arrhythmic effects of NO are PLB, NCX and RyR. At present there is little evidence showing that L-type Ca^2 +^ channels are modified by endogenous PLM. Wang et al. show effects only of PLM over-expression [50]. It seems unsurprising that the uncontrolled over-expression of a small membrane spanning protein interferes with ion channel function but it remains to be demonstrated that this occurs in cells expressing normal levels of endogenous PLM. NCX is a much more interesting possibility. According to the work of Cheung and colleagues, NO-induced phosphorylation of NCX-associated PLM would be expected to inhibit NCX [51]. Since in our studies activation of NOS protects against Ca^2 +^ overload, it seems unlikely that this would involve an inhibition of NCX.

### 4.5 Conclusions

Here we report that increased [Ca]*_i_*, as a result of heart contraction, induces synthesis of NOS-derived NO, which in turn stimulates myocardial Na/K-ATPase via a PKCε-mediated PLM phosphorylation. The resulting Na/K-ATPase stimulation plays an important role in protecting the heart against Na^+^ and Ca^2 +^ overload (via NCX) and resultant arrhythmias both at a single cell level and in a heart at physiological heart rates. Furthermore, disruption of this mechanism may result in diastolic dysfunction and hypertrophy observed in cardiomyopathies where NO synthesis is impaired such as, heart failure, uremic cardiomyopathy or sepsis.

The following are the supplementary data declared to this article.

Supplementary material

Fig. S1 PLM and PLB expression and phosphorylation. Western blots of PLM expression and phosphorylation and changes in PLM phosphorylation at Ser-63, Ser-68 and Thr-69 over 20 min of field-stimulation at 3 Hz (A). Western blots of PLB expression and phosphorylation and changes in PLB phosphorylation at Ser-16 and Thr-17 over 20 min of field-stimulation (B). The data are normalized to total expression, represent cells isolated from at least 6 individual animals and are expressed as mean ± sem (*P < 0.05).

Fig. S2 PLM phosphorylation is not CamKII or PKG dependent. Western blots showing changes in PLM expression and phosphorylation following field-stimulation (at 3 Hz, 20 min) of rat ventricular myocytes, in the presence of 2 μmol/L KN-93, 0.6 μmol/L KT-5823 or 100 μmol/L Rp-8-Br-cGMPS (A). Changes in PLM phosphorylation at Ser-63 (B) and Ser-68 (C) after 20 min of field-stimulation. The data represent cells isolated from at least 6 individual animals and are expressed as Mean ± sem (*P < 0.05 compared to 0 Hz).

Fig. S3 NO activates PKCε-isoform. Western blots showing PKCε and PKCδ translocation following 20 min of field-stimulation (A). PKCε and PKCδ cytosolic and membranous fractions (expressed as % of the total) following 20 min of field-stimulation (B)). The data represent cells isolated from 5 individual animals and are expressed as mean ± sem (*P < 0.05 compared to non-treated membrane fraction; γ*P* < 0.05 compared to non-treated cytosolic fraction).

Fig. S4 NO increases Na/K-ATPase activity by increasing its apparent K_m_, not V_max_. Effects of spermine NONOate on I_p_ in rat myocytes at intracellular Na^+^ concentration of 100 mmol/L, using perforated whole cell patch clamp technique (A). Effects of “spent” spermine NONOate on I_p_ in rat myocytes at intracellular Na^+^ concentration of 100 mmol/L, using perforated whole-cell patch clamp technique (B). The data represent cells isolated from at least 6 individual animals and are expressed as mean ± sem (*P < 0.05 compared to control). Effects of spermine NONOate on I_p_ in PLM^3SA^ myocytes at an intracellular Na^+^ concentration of 30 mmol/L, using perforated whole-cell patch clamp technique (C). The data represent cells isolated from 2 individual animals and are expressed as mean ± sem.

Fig. S5 NOS inhibition in field-stimulated rat myocytes results in elevation of Ca^2 +^ transients and sarcomere length shortening and arrhythmias. Rat myocytes were field-stimulated from quiescence at 2 Hz in the presence of 1 mmol/L l-NAME. Raw traces of Ca^2 +^ transients and sarcomere length shortening in the presence or absence of l-NAME (A). Changes in Ca^2 +^ transients following field-stimulation, in the presence or absence of l-NAME (B). Examples of arrhythmias observed during field-stimulation in the presence of l-NAME (C). The data represent cells isolated from at least 3 individual animals and are expressed as mean ± sem (*P < 0.05 compared to non-treated control).

Fig. S6 Characterization of PLM^3SA^ mice. Raw traces of the effects of forskolin on I_p_ in mouse myocytes isolated from PLM^WT^ and PLM^3SA^ animals, using perforated whole-cell patch clamp technique (A). Changes in I_p_ upon forskolin perfusion in mouse myocytes isolated from PLM^WT^ and PLM^3SA^ animals (B). Western blots showing changes in expression and phosphorylation of PLM (Ser-63, Ser-68, Ser-69), PLB (Ser-16, Thr-17), NKA α-1/2 and TnI (Ser-23/24) (C). Changes in expression of PLM and NKA α-1/2 (D). The data represent cells isolated from at least 5 individual animals and are expressed as mean ± sem (*P < 0.05 compared to control).

Fig. S7 Field-stimulation of PLM^3SA^ mouse myocytes results in elevation of diastolic Ca^2 +^ and arrhythmias. Mouse myocytes were field-stimulated from quiescence at 2 Hz for 300 s (in the absence of isoprenaline), followed by further 400 s in the presence of 1 μmol/L isoprenaline. Changes in diastolic Ca transients following field-stimulation, in the presence or absence of 1 μmol/L isoprenaline were monitored (A). Examples of arrhythmias observed during field-stimulation in PLM^3SA^ cardiac myocytes in the absence (top trace) or presence (bottom trace) of 1 μmol/L isoprenaline (B). The data represent cells isolated from at least 6 individual animals and are expressed as mean ± sem (*P < 0.05 compared to WT).

Fig. S8 PKA and NO pathways act in concert to phosphorylate PLM. Western blots showing PLM expression and phosphorylation in field-stimulated rat cardiac myocytes (3 Hz, 20 min) treated with 1 and 10 nmol/L of isoprenaline (A). Change in PLM phosphorylation at Ser-68 following 20 min of field-stimulation in the presence of 1 and 10 nmol/L isoprenaline (B). The data represent cells isolated from at least 6 individual animals and are expressed as mean ± sem (*P < 0.05 compared to 0 Hz; ψ*P* < 0.05 compared to ISO treated non-paced controls). Changes in intracellular DAF-FM fluorescence as a result of exogenously applied spermine NONO-ate (C). DAF-FM fluorescence changes are expressed as F/F_0_, representing relative increase from basal levels. Over the 1–100 μmol/L range there is a linear relationship between spermine NONO-ate concentration and cellular DAF-FM fluorescence (DAF) described by [sNO] = (DAF − 0.9182) / 0.114 (r2 = 0.8719).
