# Supplementary material for: Nitric oxide regulates cardiac intracellular Na+ and Ca2 + by modulating Na/K ATPase via PKCε and phospholemman-dependent mechanism
Source: J Mol Cell Cardiol. 2013 Aug;61:164–71. doi: 10.1016/j.yjmcc.2013.04.013 (PMC3981027; doi:10.1016/j.yjmcc.2013.04.013)
Supplement: Supplementary file 1 — Supplementary material [file mmc1.doc]

SUPPLEMENTAL INFORMATION

**Classification:** Biological Sciences - Physiology

**Title:** Nitric oxide regulates cardiac intracellular Na^+^ and Ca^2+^ by modulating Na/K ATPase via PKCε and phospholemman-dependent mechanism

**Authors:** Davor Pavlovic^1^ (PhD), Andrew R Hall^1^ (PhD), Erika J Kennington^1^ (PhD), Karen Aughton^1^, Andrii Boguslavskyii^1^ (PhD), William Fuller^2^ (PhD), Sanda Despa^3^ (PhD), Donald M. Bers^3^ (PhD) and Michael J Shattock^1^ (PhD)

**Author Affiliation:** ^1^Cardiovascular Division, King’s College London, The Rayne Institute, St Thomas’ Hospital, London, UK; ^2^Centre for Cardiovascular and Lung Biology, University of Dundee, Dundee, UK; ^3^Department of Pharmacology, University of California Davis, CA, USA.

**Corresponding author:** Professor Michael Shattock, Cardiac Physiology, The Rayne Institute, St Thomas’ Hospital, London SE1 7EH. Tel:+44(0)2071880945 Fax: +44(0)2071880970 e-mail: michael.shattock@kcl.ac.uk

**1. DETAILED METHODS**

**1.1. Animals and cell isolation**

Both PLM^KO^ and PLM^3SA^ mice were all created on the same C57BL/6J background. PLM^3SA^ mice were generated by Genoway as follows: The *Fxyd-1* point mutation Knock-in mouse model was generated by homologous recombination in embryonic stem cells (ES) using a targeting vector. The targeting strategy leads to the disruption of the murine *Fxyd-1* gene by insertion of S63A, S68A and S69A point mutations in exons 6 and 7 resulting in the expression of a constitutively unphosphorylated FXYD-1 protein. The mutated *Fxyd-1* gene is expressed under the control of the endogenous *FXYD-1* promoter. After electroporation of the targeting vector into 129Sv ES cells, G418 resistant ES cell clones were screened by PCR and Southern Blot for homologous recombination event.  Recombined ES cell clones were thereafter injected into C57BL/6J derived blastocysts to generate chimeric mice. Germline transmission of the mutated *Fxyd-1* allele and *in vivo* deletion of the loxP-neomycin-loxP selection cassette were then assessed by breeding of chimeras with Cre-expressing deleter C57BL/6J mice. Knock-in heterozygous mice were characterized by PCR and Southern Blot. PLM KO mice were compared with age-matched wild type littermates. PLM KO mice were generated in the Transgenic Facility at the University of Virginia (Charlottesville, VA, USA; 8, 9). Mice were congenic on a pure C57B/6 background. Heterozygous breeding pairs were used to generate FXYD1 KO and WT littermates. Mice of 3 months of age were used in this study and all have received humane care in accordance with “Guidance on the Operation of the Animals (Scientific Procedures) Act of 1986” published by HM Stationery Office, London UK and the Guide for the Care and Use of Laboratory Animals published by the U.S. National Institutes of Health (NIH Publication No. 85–23, revised 1996).

Adult ventricular myocytes were isolated from the hearts of adult male Wistar rats (200-250 g, B&K Universal) and adult male C57Bl/6 mice (15 weeks of age) by standard collagenase enzymatic digestion . Animals were anesthetized with sodium pentobarbital in combination with sodium heparin (200 mg/kg and 200 IU/kg, respectively). In the majority of experiments, myocytes were used two hours post-isolation. Experimental data obtained in Animals were maintained humanely in compliance with the "Principles of Laboratory Animal Care" formulated by the National Society for Medical Research and the Guide for Care and Use of Laboratory Animals prepared by the National Academy of Sciences and published by the National Institutes of Health (NIH Pub. No. 85-23, revised 1985). All animal protocols were approved both by the local King's College Ethical Review Process Com­mittee and by the UK Government Home Office (Animals Scientific Procedures Group).

**1.2. Contractility, Ca^2+^ transients and endogenous NO production in ARVM.**

Sarcomere shortening, Ca transients and endogenous NO production were measured using an integrated contractility/photometry system (IonOptix Corporation, USA). Adult rat ventricular myocytes were perfused with a standard Tyrode’s solution (containing (mmol/L) NaCl 130, KCl 5.4, HEPES 11.8, MgCl_2_ 0.5, CaCl_2_ 1.8, and glucose 10 (pH 7.4 at 35˚C) in an open-perfusion chamber mounted on the stage of an inverted microscope. Cells were field stimulated at 40 V (pulse width 5 msec) at a frequency of 2 or 3 Hz. Changes in sarcomere length were recorded from individually selected myocytes using IonOptix software (sampled at 240 Hz).

Endogenous NO production was measured using the NO sensitive fluorescent dye 4,5-diaminofluorescein (DAF-FM). Myocytes were incubated in Tyrode’s solution containing 5μM DAF-FM diacetate, 100 μmol/L L-arginine and 1mM probenecid (Sigma, UK) for 40 minutes at room temperature before myocytes were briefly washed in normal Tyrode’s solution. DAF-FM fluorescence was excited at 480 nm and the emitted cellular fluorescence recorded at 540nm. Changes in DAF-FM intensity (F) in each experiment were normalized to the level of fluorescence recorded prior to stimulation (F_0_). Changes in intracellular NO are expressed as F/F_0_, representing relative increase from basal levels. In preliminary characterisation experiments, DAF-FM fluorescence was calibrated in intact cells with respect to a range of exogenously applied spermine NONOate (sNO) concentrations. Over the range 1-125 µmol/L there was a linear relationship (Figure S8C) between sNO concentration (in µmol/L) and cellular DAF-FM fluorescence (DAF) described by [sNO] = (DAF-0.9182)/0.114 (r2 = 0.8719).

Endogenous Ca transients were measured with Fura-2-AM (Invitrogen, US). Briefly, mouse ventricular myocytes were loaded with Fura-2-AM for 20 min and [Ca]_i_ measured throughout the pacing period (2 Hz). The normal Tyrode’s solution contained (in mmol/L): 140 NaCl, 4 KCl, 1 MgCl2, 1 CaCl2, 10 HEPES, and 10 glucose (pH 7.4). Dual excitation measurements (at 340 and 380 nm; F340 and F380) were performed and emission light was collected at 510 nm. All the measurements were done at 37 degrees C.

**1.3. Field stimulation of ARVM**

Myocytes were plated, in M199 culture medium supplemented with 100 μmol/L L-arginine (Sigma), onto laminin-coated four well culture dishes (IonOptix Corporation, USA) immediately post-isolation. After 2h incubation, myocytes were electrically paced using a C-pace multi-channel stimulator (Ionoptix Corp., US) for up to 20 min at 40 V and 3 Hz after which the extracellular solution was removed and the myocytes lysed in sample buffer (250 mmol/L Tris, 0.2 mmol/L EDTA, 5% mercaptoethanol, 4% SDS, 20% glycerol, 0.02% bromphenol blue, pH 6.8) before being loaded and separated by SDS-PAGE as described previously. For inhibitor experiments, cells were pre-incubated for 30 min in the presence of either L-NAME (1 mmol/L), H-89 (2 μmol/L), Bis (2 μmol/L), ODQ (1 μmol/L), Rp-8-Br-cGMPS (100 μmol/L) or KT5823 (1 μmol/L, 0.6 μmol/L). PMA (300 nmol/L) and Forskolin (50 μmol/L) were used as positive controls.

For PKC translocation studies, adult rat ventricular myocytes were electrically paced for 20 min (20 V and 3 Hz) and exposed to either 300 nmol/L of PMA, 1 mmol/L of L-NAME, 10 mmol/L of EGTA or 1 μmol/L of Bis (for 20 min). Cells were then washed with PBS and harvested in chilled lysis buffer (in mmol/L) 50 Tris-HCl, 5 EGTA, 2 EDTA; 100 NaF, 1 AEBSF, 5 DTT, with 10 mg/ml leupeptin, 10 mg/ml pepstatin A, 0.05% digitonin, pH 7.5. After vortexing for 5 min, cytosolic and membrane fractions were separated by centrifugation (10,000 g for 5 min at 4°C). Cytosolic and membrane fractions were resuspended in equal volumes of SDS-sample buffer and analyzed by western blotting.

**1.4. Western blotting**

Samples were lysed in sample buffer (2 mmol/L Tris-HCl, 0.2 mmol/L EDTA, 20 mmol/L DTT, 4% SDS, 10% glycerol, 0.04% bromphenol blue, pH 8.0) before being loaded and separated by weight using SDS-PAGE on a 15% polyacrylamide gel. Separated proteins were then transferred on to a PVDF membrane (GE Healthcare UK) which were blocked overnight in PBS containing 5% milk and 0.1% Tween-20 at 4°C. Membranes were subsequently probed with PLM phospho-specific antibodies diluted in PBS containing 5% milk and 0.1% Tween-20 for 1 hour at room temperature (see Fuller *et al* 2009^5^). After multiple washes, membranes were probed with secondary antibodies for an hour before again being repeatedly washed in PBS buffer with 0.1% Tween-20. Membranes were finally incubated with enhanced chemiluminescence reagent (ECL, Amersham) and visualized on Hyperfilm (Amersham, UK). Relative PLM residue phosphorylation was quantified through film-scanning and densitometry using Quantity One software (Biorad).

**1.5. Na/K ATPase assay**

Adult rat ventricular myocytes were plated in M199 culture medium supplemented with 100 μmol/L L-arginine (Sigma, USA) onto laminin-coated four well culture dishes (IonOptix Corporation, USA) immediately post-isolation. After 2 hr incubation, myocytes were electrically paced using a C-pace multi-channel stimulator (Ionoptix Corp., US) for 20 min at 40 V and 3 Hz. Pacing was performed in the presence or absence of L-NAME (1 mmol/L), L-NIO (1 μmol/L and 100 μmol/L) and Bis (2 μmol/L) after which the extracellular solution was removed and myocytes collected in SET solution (1 mmol/L EDTA, 20 mmol/L Tris, pH 7.5; supplemented with protease inhibitors coctail I (Merck, USA) and phospatase inhibitor coctail III (Sigma, USA)). Membranes were lysed by 20 seconds of sonication and Na/K ATPase assay based on Baginsky method was performed as previously described. Protein concentrations in each fraction were determined using the method of Bradford.

**1.6. Electrophysiology**

Adult ventricular myocytes isolated from both rat and mice were voltage-clamped and Na/K pump current (*I*_p_) was recorded at 35˚C using the whole-cell perforated-patch technique as described elsewhere. Myocytes were studied under whole-cell voltage-clamp using electrodes which had resistances of 1-2 MΩ when filled with when filled with (in mmol/L) 110 CsCH_3_O_3_S, 15 NaCH_3_O_3_, 15 NaCl, 8 CsCl, 1 MgCl_2_, 10 HEPES, pH 7.2. External solution was (in mmol/L) 140 NaCl, 1 MgCl_2_, 2 NiCl_2_, 1 BaCl_2_, 5 KCl, 10 glucose, 10 HEPES, pH 7.4 at 35°C unless otherwise stated. Cell capacitance was measured from the capacitance transient generated on application of a voltage step from the holding potential of -90mV to -80 mV. Current generated was recorded via an Axopatch 200 A amplifier and pClamp10 software (Molecular Devices, California, USA). Cells were incubated for 1h or acutely treated with NO donor, spermine NONOate. All experiments were carried out in subdued light and with the microscope illumination switched off in order to limit the degradation of the NO donors used and/or photolabile nitrosylated protein adducts.

**1.7. Isolated heart perfusion and arrhythmia analysis**

Hearts isolated from adult male Wistar rats (200-250 g, B&K Universal) and WT and PLM^3SA^ mice, were Langendorff-perfused with Krebs-Henseleit (KH) bicarbonate buffer (37^o^C) containing (in mmol/L) NaCl 118, NaHCO_3_ 25.0, KCl 4.7, MgCl_2_ 1.2, KH2PO4 1.18, glucose 11.1, CaCl_2_ 1.4 (pH 7.4 when gassed with 95%O_2_:5%CO_2_). Hearts were perfused at a constant pressure of 73mmHg using a feedback system (STH Pump Controller, AD Instruments).

In initial experiments, rat hearts were paced at 300 bpm or 600 bpm and mice hearts at 550 and 800 bpm (5msec pulse, 1.5 times threshold) via a unipolar electrode inserted into the base of the left ventricle with reference to the metal aortic cannula and monitored for arrhythmias using heart rate variability software. In separate experiments in rat hearts, VF threshold was determined using a protocol adapted from Zaugg *et al* . In these experiments a modified KH buffer was used in which K was reduced to 2.8 mmol/L and Ca was raised to 1.8 mmol/L. Hearts were paced via bipolar electrodes inserted into the epicardial surface of the base of the left-ventricle at a constant distance separation (5 mm) and depth (2 mm). Hearts were then paced at a constant current (5 msec pulse, 1.5 times threshold) at a basal rate of either 300 bpm (control) or 600 bpm (rapid pacing). Basal pacing was terminated and 100 ms later a train of 10 pulses (100 Hz: train duration 100 ms) was imposed before allowing the heart to beat spontaneously and the return to sinus rhythm monitored. The train of pulses was timed to overlay the vulnerable window and was initially imposed with a sub-threshold current of 0.5 mA. On return to sinus rhythm, basal pacing was resumed and the pulse-train current was increased in 0.5 mA steps and the protocol repeated until VF was induced. This protocol was repeated 3 times with hearts defibrillated between protocols and the average current required to induce VF determined for each heart.

**1.8. Statistical analysis**

Quantitative data are shown as mean±standard error of the mean (SEM). Differences between experimental groups were tested by one-way ANOVA followed by a Bonferroni post-hoc test or by paired or unpaired T-tests. In arrhythmia studies, VF incidence was compared using Fisher’s Exact Test and contingency tables. In experiments measuring VF threshold, pilot experiments showed that within heart variability was normally distributed (as assessed by the Kolmogorov-Smirnov test for normality), however, as previously shown by others , variation in VF threshold between hearts was logarithmically distributed. Log_10_ VF thresholds were therefore compared by one-way ANOVA followed by a post-hoc Student Newman-Keuls test. Differences were considered significant at p<0.05.

**2. SI REFERENCES**

[1] Bell JR, Kennington E, Fuller W, Dighe K, Donoghue P, Clark JE, et al. Characterization of the phospholemman knockout mouse heart: depressed left ventricular function with increased Na-K-ATPase activity. American journal of physiology Heart and circulatory physiology. 2008;294:H613-21.

[2] Pavlovic D, McLatchie LM, Shattock MJ. The rate of loss of T-tubules in cultured adult ventricular myocytes is species dependent. Exp Physiol. 2010;95:518-27.

[3] Fuller W, Howie J, McLatchie LM, Weber RJ, Hastie CJ, Burness K, et al. FXYD1 phosphorylation in vitro and in adult rat cardiac myocytes: threonine 69 is a novel substrate for protein kinase C. American journal of physiology Cell physiology. 2009;296:C1346-55.

[4] Fuller W, Eaton P, Bell JR, Shattock MJ. Ischemia-induced phosphorylation of phospholemman directly activates rat cardiac Na/K-ATPase. FASEB J. 2004;18:197-9.

[5] Pavlovic D, Fuller W, Shattock MJ. The intracellular region of FXYD1 is sufficient to regulate cardiac Na/K ATPase. FASEB J. 2007;21:1539-46.

[6] Zaugg CE, Wu ST, Lee RJ, Wikman-Coffelt J, Parmley WW. Intracellular Ca2+ handling and vulnerability to ventricular fibrillation in spontaneously hypertensive rats. Hypertension. 1997;30:461-7.

**3. SI FIGURES AND FIGURE LEGENDS**

**Figure S1:** **PLM and PLB expression and phosphorylation**. Western blots of PLM expression and phosphorylation and changes in PLM phosphorylation at Ser-63, Ser-68 and Thr-69 over 20min of field-stimulation at 3Hz (**A)**. Western blots of PLB expression and phosphorylation and changes in PLB phosphorylation at Ser-16 and Thr-17 over 20min of field-stimulation (**B)**. The data are normalized to total expression, represent cells isolated from at least 6 individual animals and are expressed as Mean±sem (**P* < 0.05).

**Figure S2: PLM phosphorylation is not CamKII or PKG dependent.** Western blots showing changes in PLM expression and phosphorylation following field-stimulation (at 3 Hz, 20 min) of rat ventricular myocytes, in the presence of 2 μmol/L KN-93, 0.6 μmol/L KT-5823 or 100 μmol/L Rp-8-Br-cGMPS (**A**). Changes in PLM phosphorylation at Ser-63 (**B)** and Ser-68 (**C)** after 20 min of field-stimulation. The data represents cells isolated from at least 6 individual animals and are expressed as Mean±sem (**P* < 0.05 compared to 0 Hz).

**Figure S3:** **NO activates PKCε-isoform.** Western blots showing PKCε and PKCδ translocation following 20 min of field-stimulation (**A**). PKCε and PKCδ cytosolic and membranous fractions (expressed as % of total following 20 minutes of field-stimulation (**B**). The data represent cells isolated from 5 individual animals and are expressed as mean±sem (**P* < 0.05 compared to non treated membrane fraction; **^γ^***P* < 0.05 compared to non treated cytosolic fraction).

**Figure S4:** **NO increases Na/K-ATPase activity by increasing its apparent K_m_, not V_max_.** Effects of spermine NONOate on I_p_ in rat myocytes at intracellular Na^+^ concentration of 100 mmol/L, using perforated whole cell patch clamp technique (**A**). Effects of “spent” spermine NONOate on I_p_ in rat myocytes at intracellular Na^+^ concentration of 100 mmol/L, using perforated whole-cell patch clamp technique (**B**). The data represent cells isolated from at least 6 individual animals and are expressed as Mean±sem (**P* < 0.05 compared to control). Effects of spermine NONOate on I_p_ in PLM^3SA^ myocytes at intracellular Na^+^ concentration of 30 mmol/L, using perforated whole-cell patch clamp technique (**C**). The data represent cells isolated from 2 individual animals and are expressed as Mean±sem.

**Figure S5:** **NOS inhibition in field-stimulated rat myocytes results in elevation of Ca^2+^ transients and sarcomere length shortening and arrhythmias.** Rat myocytes were field-stimulated from quiescence at 2 Hz in the presence of 1 mmol/L L-NAME. Raw traces of Ca^2+^ transients and sarcomere length shortening in the presence or absence of L-NAME **(A).** Changes in Ca^2+^ transients following field-stimulation, in the presence or absence of L-NAME **(B).** Examples of arrhythmias observed during field-stimulation in in the presence of L-NAME (**C**). The data represent cells isolated from at least 3 individual animals and are expressed as mean±sem. (**P* < 0.05 compared to non-treated control)

**Figure S6:** **Characterization of PLM^3SA^ mice.** Raw traces of the effects of forskolin on I_p_ in mouse myocytes isolated from PLM^WT^ and PLM^3SA^ animals, using perforated whole-cell patch clamp technique (**A**). Changes in I_p_ upon forskolin perfusion in mouse myocytes isolated from PLM^WT^ and PLM^3SA^ animals (**B**). Western blots showing changes in expression and phosphorylation of PLM (Ser-63, Ser-68, Ser-69), PLB (Ser-16, Thr-17), NKA α-1/2 and TnI (Ser-23/24) **(C)**. Changes in expression of PLM and NKA α-1/2 **(D)**. The data represent cells isolated from at least 5 individual animals and are expressed as mean±sem (**P* < 0.05 compared to control).

**Figure S7.** **Field-stimulation of PLM^3SA^ mouse myocytes results in elevation of diastolic Ca^2+^ and arrhythmias.** Mouse myocytes were field-stimulated from quiescence at 2 Hz for 300 seconds (in the absence of isoprenaline), followed by further 400 seconds in the presence of 1 μmol/L isoprenaline. Changes in diastolic Ca transients following field-stimulation, in the presence or absence of 1 μmol/L isoprenaline were monitored **(A).** Examples of arrhythmias observed during field-stimulation in PLM^3SA^ cardiac myocytes in the absence (top trace) or presence (bottom trace) of 1 μmol/L isoprenaline (**B**). The data represent cells isolated from at least 6 individual animals and are expressed as mean±sem (**P* < 0.05 compared to WT).

**Figure S8:** **PKA and NO pathways act in concert to phosphorylate PLM.** Western blots showing PLM expression and phosphorylation in field-stimulated rat cardiac myocytes (3 Hz, 20 min) treated with 1 and 10 nmol/L of isoprenaline (**A**). Change in PLM phosphorylation at Ser-68 following 20 min of field-stimulation in the presence of 1 and 10 nmol/L isoprenaline **(B).** The data represent cells isolated from at least 6 individual animals and are expressed as Mean±sem (**P* < 0.05 compared to 0 Hz; ^^*P* < 0.05 compared to ISO treated non-paced controls). Changes in intracellular DAF-FM fluorescence as a result of of exogenously applied spermine NONO-ate **(C).** DAF-FM fluorescence changes are expressed as F/F_0_, representing relative increase from basal levels. Over the range 1-100 µmol/L there is a linear relationship between spermine NONO-ate concentration and cellular DAF-FM fluorescence (DAF) described by [sNO] = (DAF-0.9182)/0.114 (r2 = 0.8719).
