# Supplementary material for: Nitric oxide regulates cardiac intracellular Na+ and Ca2 + by modulating Na/K ATPase via PKCε and phospholemman-dependent mechanism
Source: J Mol Cell Cardiol. 2013 Aug;61:164–71. doi: 10.1016/j.yjmcc.2013.04.013 (PMC3981027; doi:10.1016/j.yjmcc.2013.04.013)
Supplement: Fig. S1 — PLM and PLB expression and phosphorylation. Western blots of PLM expression and phosphorylation and changes in PLM phosphorylation at Ser-63, Ser-68 and Thr-69 over 20 min of field-stimulation at 3 Hz (A). Western blots of PLB expression and phosphorylation and changes in PLB phosphorylation at Ser-16 and Thr-17 over 20 min of field-stimulation (B). The data are normalized to total expression, represent cells isolated from at least 6 individual animals and are expressed as mean ± sem (*P < 0.05). [file mmc2.ppt]

## Slide 1
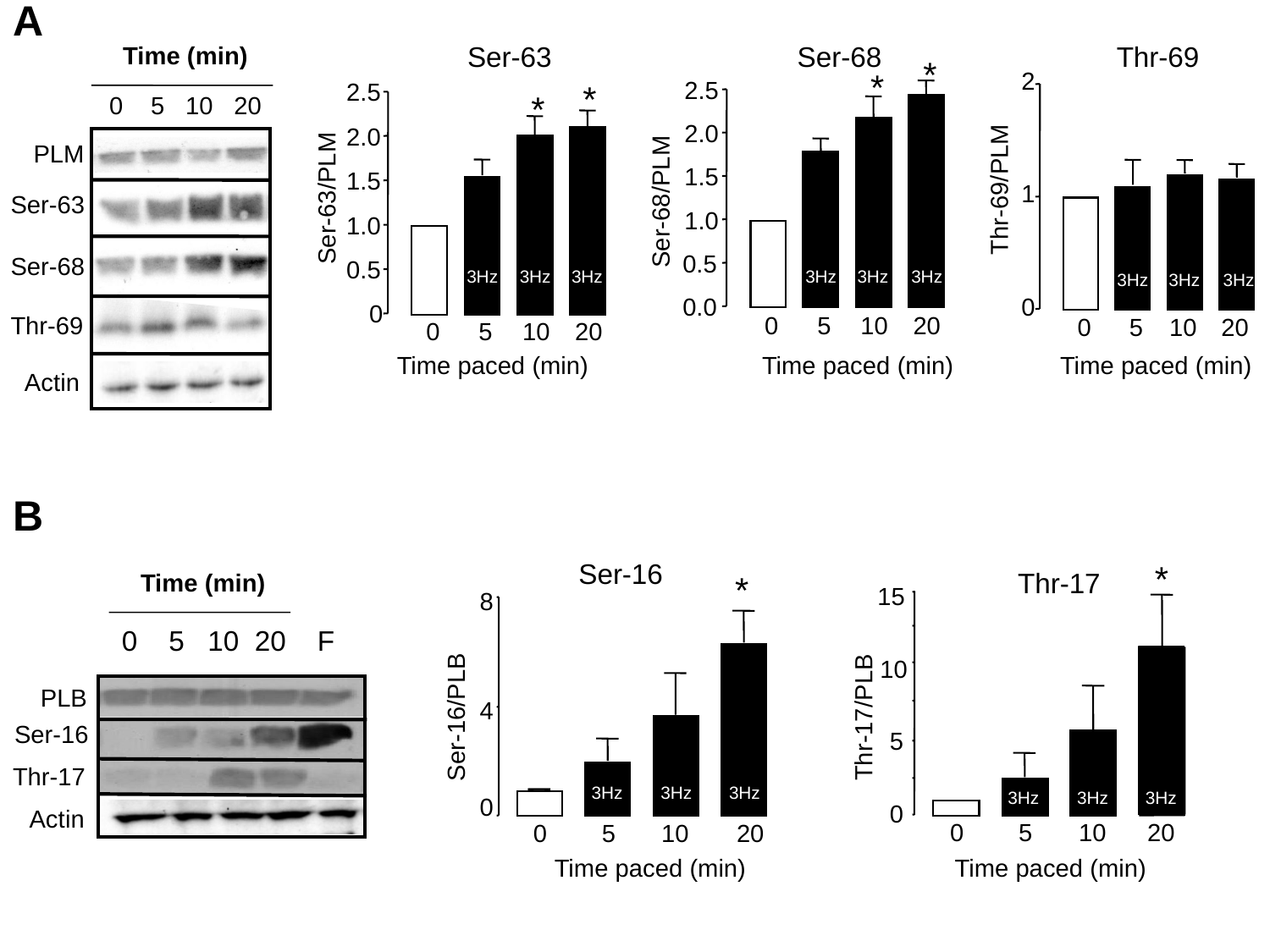

A
Time (min)
Ser-63
Ser-68
Thr-69
*
*
2
2.5
2.5
*
*
 0 5 10 20
2.0
2.0
PLM
1.5
1.5
Thr-69/PLM
1
Ser-63/PLM
Ser-68/PLM
Ser-63
1.0
1.0
0.5
Ser-68
0.5
0
0.0
0
Thr-69
0
5
10
20
0
5
10
20
0
5
10
20
Time paced (min)
Time paced (min)
Time paced (min)
Actin
B
Ser-16
*
Time (min)
Thr-17
*
15
8
0 5 10 20 F
10
PLB
4
Ser-16/PLB
Thr-17/PLB
Ser-16
5
Thr-17
0
0
Actin
0
5
10
20
0
5
10
20
Time paced (min)
Time paced (min)
3Hz
3Hz
3Hz
3Hz
3Hz
3Hz
3Hz
3Hz
3Hz
3Hz
3Hz
3Hz
3Hz
3Hz
3Hz
3Hz
