# Supplementary material for: Nitric oxide regulates cardiac intracellular Na+ and Ca2 + by modulating Na/K ATPase via PKCε and phospholemman-dependent mechanism
Source: J Mol Cell Cardiol. 2013 Aug;61:164–71. doi: 10.1016/j.yjmcc.2013.04.013 (PMC3981027; doi:10.1016/j.yjmcc.2013.04.013)
Supplement: Fig. S2 — PLM phosphorylation is not CamKII or PKG dependent. Western blots showing changes in PLM expression and phosphorylation following field-stimulation (at 3 Hz, 20 min) of rat ventricular myocytes, in the presence of 2 μmol/L KN-93, 0.6 μmol/L KT-5823 or 100 μmol/L Rp-8-Br-cGMPS (A). Changes in PLM phosphorylation at Ser-63 (B) and Ser-68 (C) after 20 min of field-stimulation. The data represent cells isolated from at least 6 individual animals and are expressed as Mean ± sem (*P < 0.05 compared to 0 Hz). [file mmc3.ppt]

## Slide 1
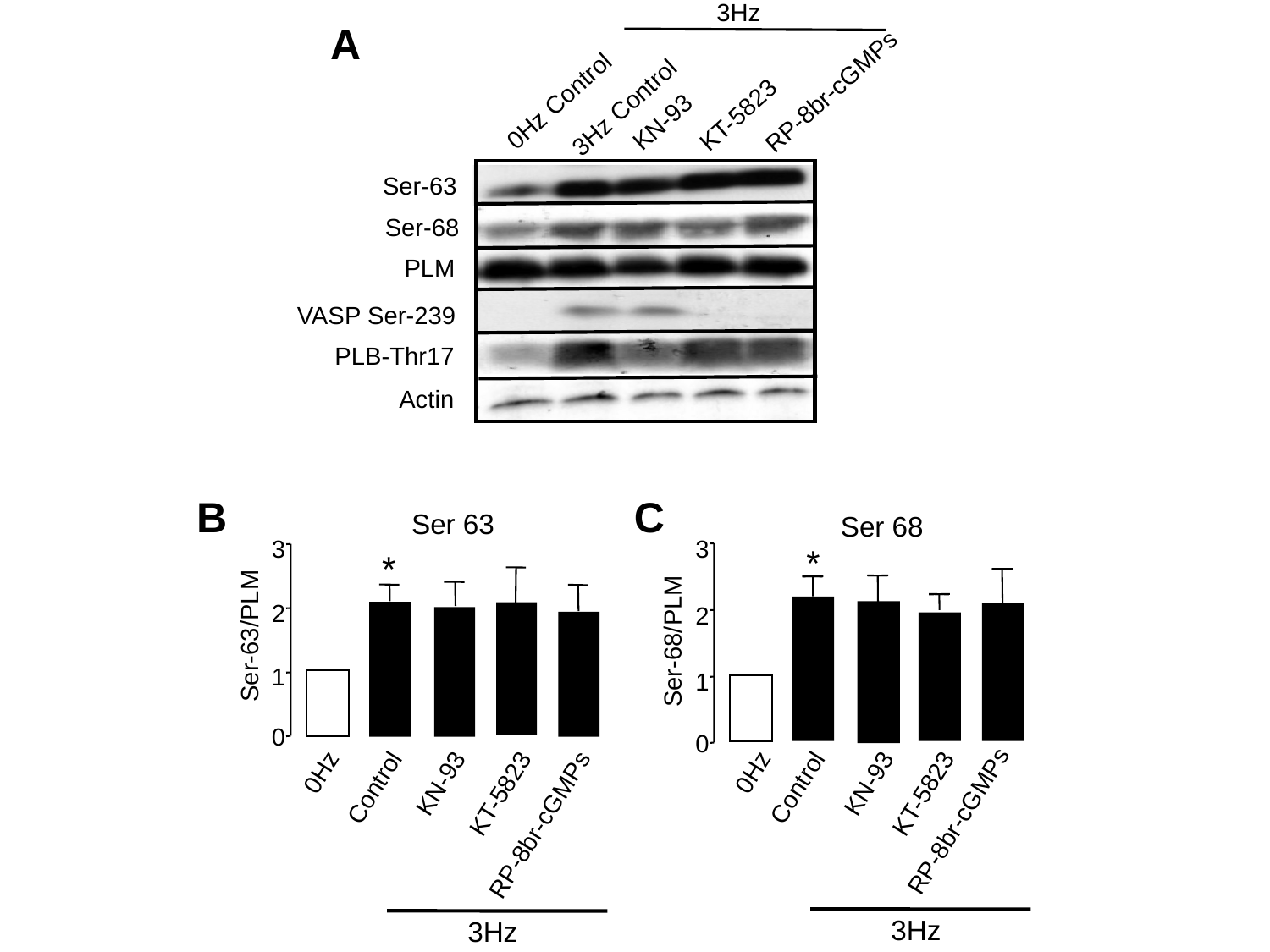

3Hz
A
KN-93
KT-5823
RP-8br-cGMPs
0Hz Control
3Hz Control
Ser-63
Ser-68
PLM
VASP Ser-239
PLB-Thr17
Actin
B
C
Ser 63
Ser 68
3
2
1
0
3
*
*
2
Ser-63/PLM
Ser-68/PLM
1
0
0Hz
Control
KN-93
0Hz
RP-8br-cGMPs
Control
KN-93
KT-5823
RP-8br-cGMPs
KT-5823
3Hz
3Hz
