# Supplementary figures and images for: Nitric oxide regulates cardiac intracellular Na+ and Ca2 + by modulating Na/K ATPase via PKCε and phospholemman-dependent mechanism
Source: J Mol Cell Cardiol. 2013 Aug;61:164–71. doi: 10.1016/j.yjmcc.2013.04.013 (PMC3981027; doi:10.1016/j.yjmcc.2013.04.013)

## Slide 1
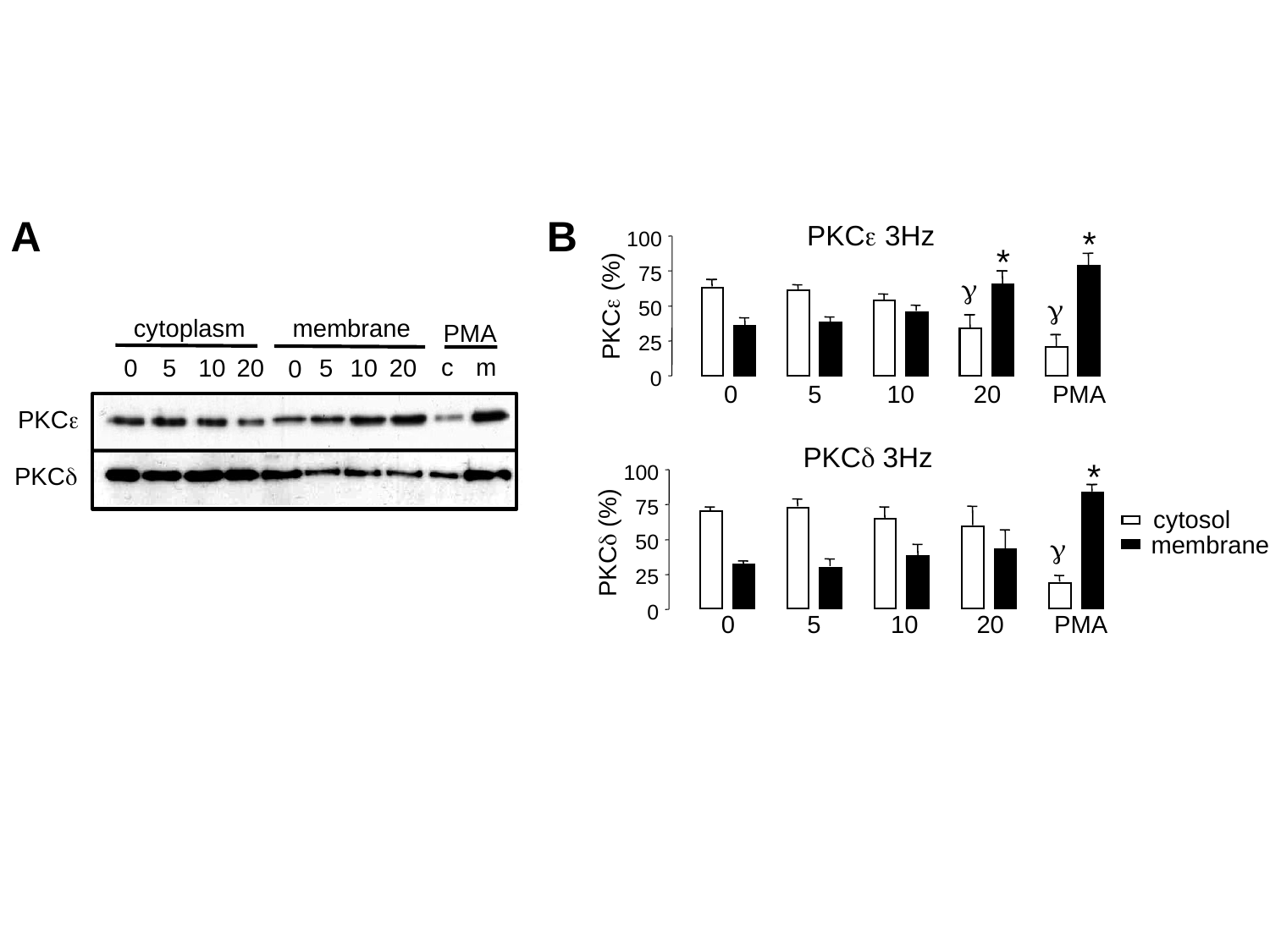

A
B
PKCε 3Hz
*
100
*
75
γ
PKCε (%)
γ
50
25
0
0
5
10
20
PMA
PKCδ 3Hz
*
100
75
cytosol
membrane
PKCδ (%)
50
γ
25
0
0
5
10
20
PMA
cytoplasm
membrane
PMA
c
m
0
5
10
20
5
10
20
0
PKCε
PKCδ

Supplement: Fig. S3 — NO activates PKCε-isoform. Western blots showing PKCε and PKCδ translocation following 20 min of field-stimulation (A). PKCε and PKCδ cytosolic and membranous fractions (expressed as % of the total) following 20 min of field-stimulation (B)). The data represent cells isolated from 5 individual animals and are expressed as mean ± sem (*P < 0.05 compared to non-treated membrane fraction; γP < 0.05 compared to non-treated cytosolic fraction). [file mmc4.ppt]

## Slide 1
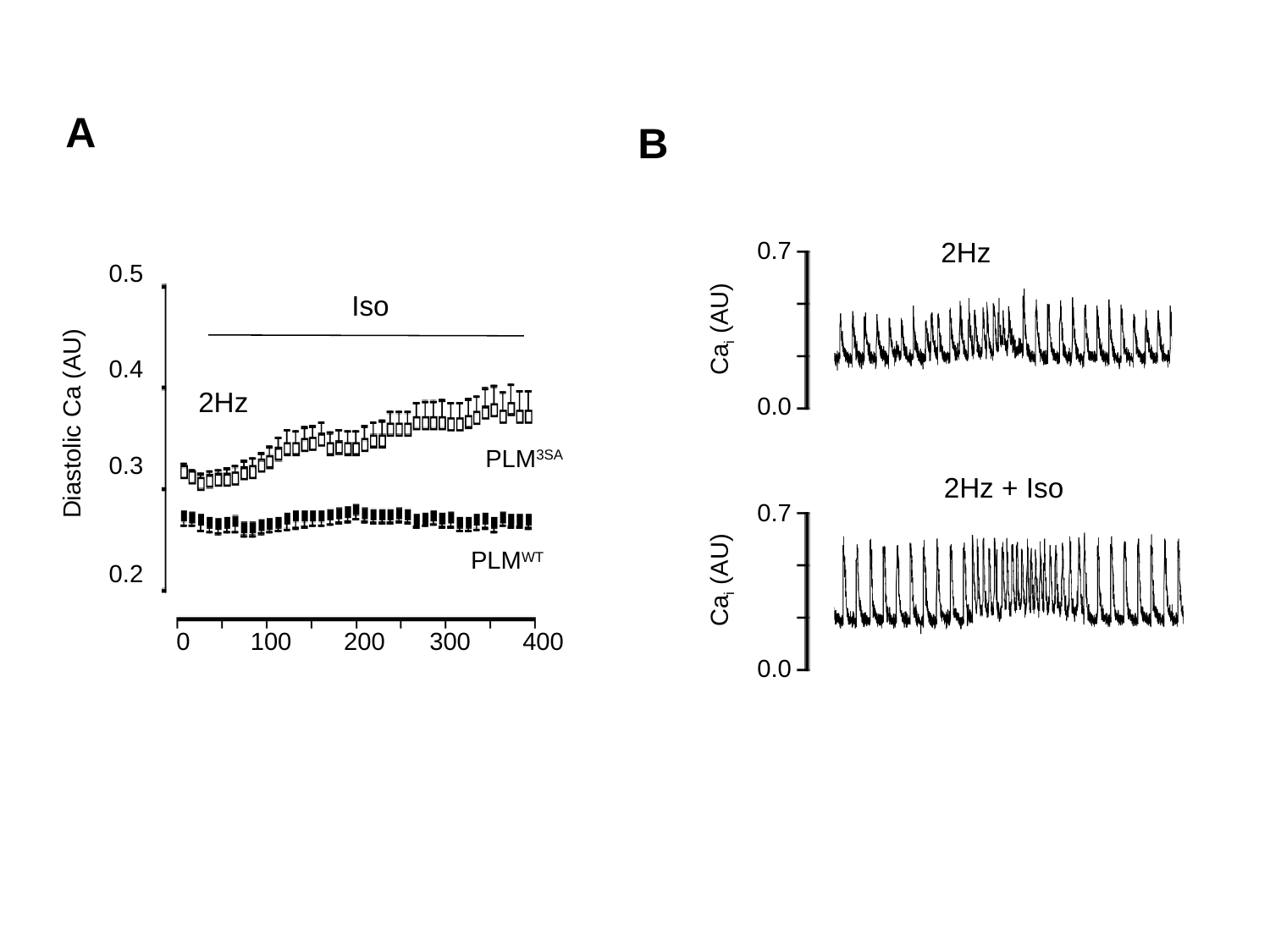

A
B
2Hz
0.7
0.0
0.5
Cai (AU)
Iso
0.4
2Hz
Diastolic Ca (AU)
PLM3SA
0.3
2Hz + Iso
0.7
0.0
Cai (AU)
PLMWT
0.2
0
100
200
300
400

Supplement: Fig. S7 — Field-stimulation of PLM3SA mouse myocytes results in elevation of diastolic Ca2 + and arrhythmias. Mouse myocytes were field-stimulated from quiescence at 2 Hz for 300 s (in the absence of isoprenaline), followed by further 400 s in the presence of 1 μmol/L isoprenaline. Changes in diastolic Ca transients following field-stimulation, in the presence or absence of 1 μmol/L isoprenaline were monitored (A). Examples of arrhythmias observed during field-stimulation in PLM3SA cardiac myocytes in the absence (top trace) or presence (bottom trace) of 1 μmol/L isoprenaline (B). The data represent cells isolated from at least 6 individual animals and are expressed as mean ± sem (*P < 0.05 compared to WT). [file mmc8.ppt]

## Slide 1
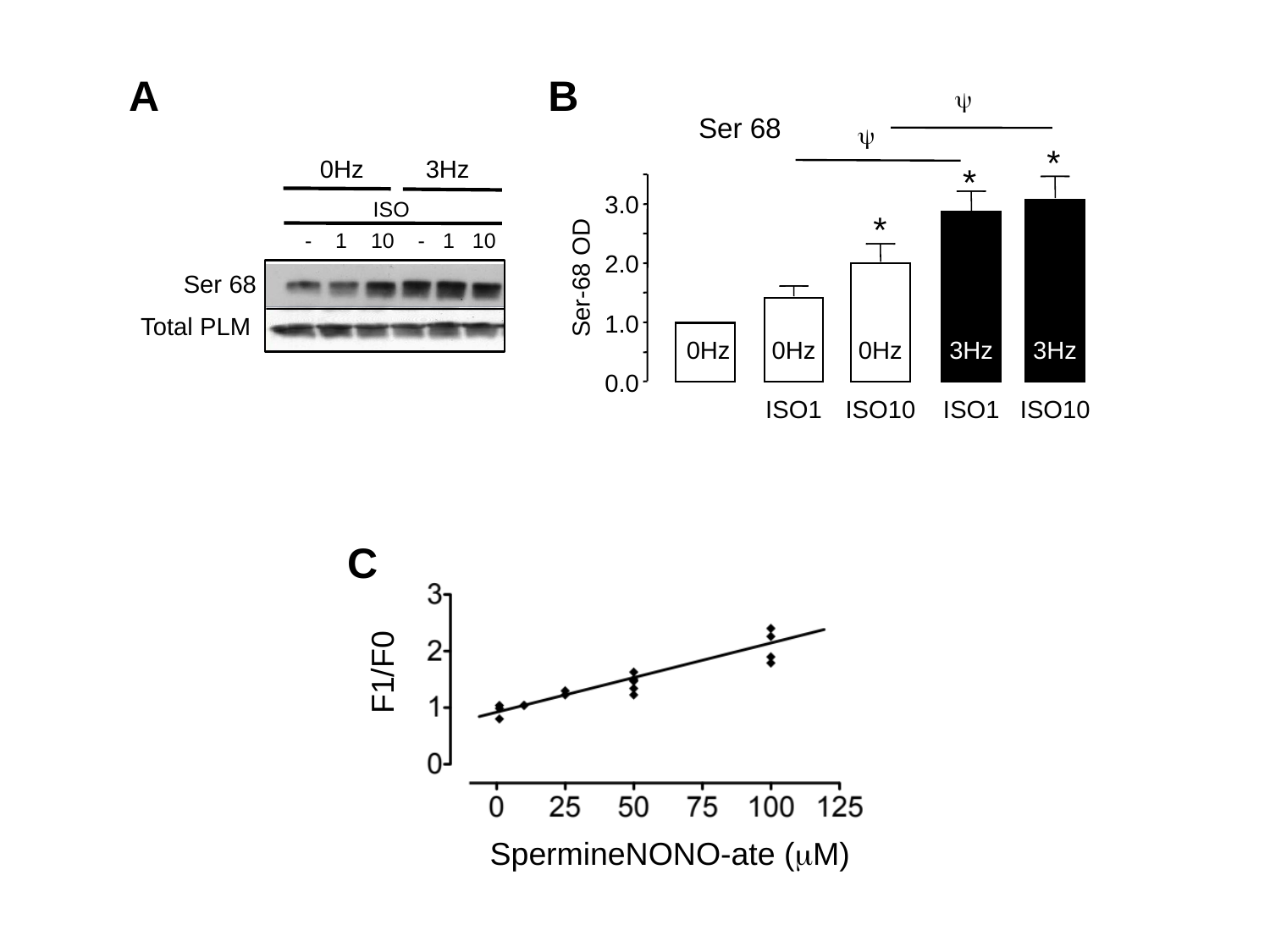

A
B

Ser 68

*
*
3.0
*
2.0
1.0
0Hz
0Hz
ISO1
0Hz
ISO10
3Hz
ISO1
3Hz
ISO10
0.0
0Hz
3Hz
ISO
 - 1 10 - 1 10
Ser-68 OD
Ser 68
Total PLM
C
F1/F0
SpermineNONO-ate (μM)

Supplement: Fig. S8 — PKA and NO pathways act in concert to phosphorylate PLM. Western blots showing PLM expression and phosphorylation in field-stimulated rat cardiac myocytes (3 Hz, 20 min) treated with 1 and 10 nmol/L of isoprenaline (A). Change in PLM phosphorylation at Ser-68 following 20 min of field-stimulation in the presence of 1 and 10 nmol/L isoprenaline (B). The data represent cells isolated from at least 6 individual animals and are expressed as mean ± sem (*P < 0.05 compared to 0 Hz; ψP < 0.05 compared to ISO treated non-paced controls). Changes in intracellular DAF-FM fluorescence as a result of exogenously applied spermine NONO-ate (C). DAF-FM fluorescence changes are expressed as F/F0, representing relative increase from basal levels. Over the 1–100 μmol/L range there is a linear relationship between spermine NONO-ate concentration and cellular DAF-FM fluorescence (DAF) described by [sNO] = (DAF − 0.9182) / 0.114 (r2 = 0.8719). [file mmc9.ppt]
