# Supplementary material for: Nitric oxide regulates cardiac intracellular Na+ and Ca2 + by modulating Na/K ATPase via PKCε and phospholemman-dependent mechanism
Source: J Mol Cell Cardiol. 2013 Aug;61:164–71. doi: 10.1016/j.yjmcc.2013.04.013 (PMC3981027; doi:10.1016/j.yjmcc.2013.04.013)
Supplement: Fig. S4 — NO increases Na/K-ATPase activity by increasing its apparent Km, not Vmax. Effects of spermine NONOate on Ip in rat myocytes at intracellular Na+ concentration of 100 mmol/L, using perforated whole cell patch clamp technique (A). Effects of “spent” spermine NONOate on Ip in rat myocytes at intracellular Na+ concentration of 100 mmol/L, using perforated whole-cell patch clamp technique (B). The data represent cells isolated from at least 6 individual animals and are expressed as mean ± sem (*P < 0.05 compared to control). Effects of spermine NONOate on Ip in PLM3SA myocytes at an intracellular Na+ concentration of 30 mmol/L, using perforated whole-cell patch clamp technique (C). The data represent cells isolated from 2 individual animals and are expressed as mean ± sem. [file mmc5.ppt]

## Slide 1
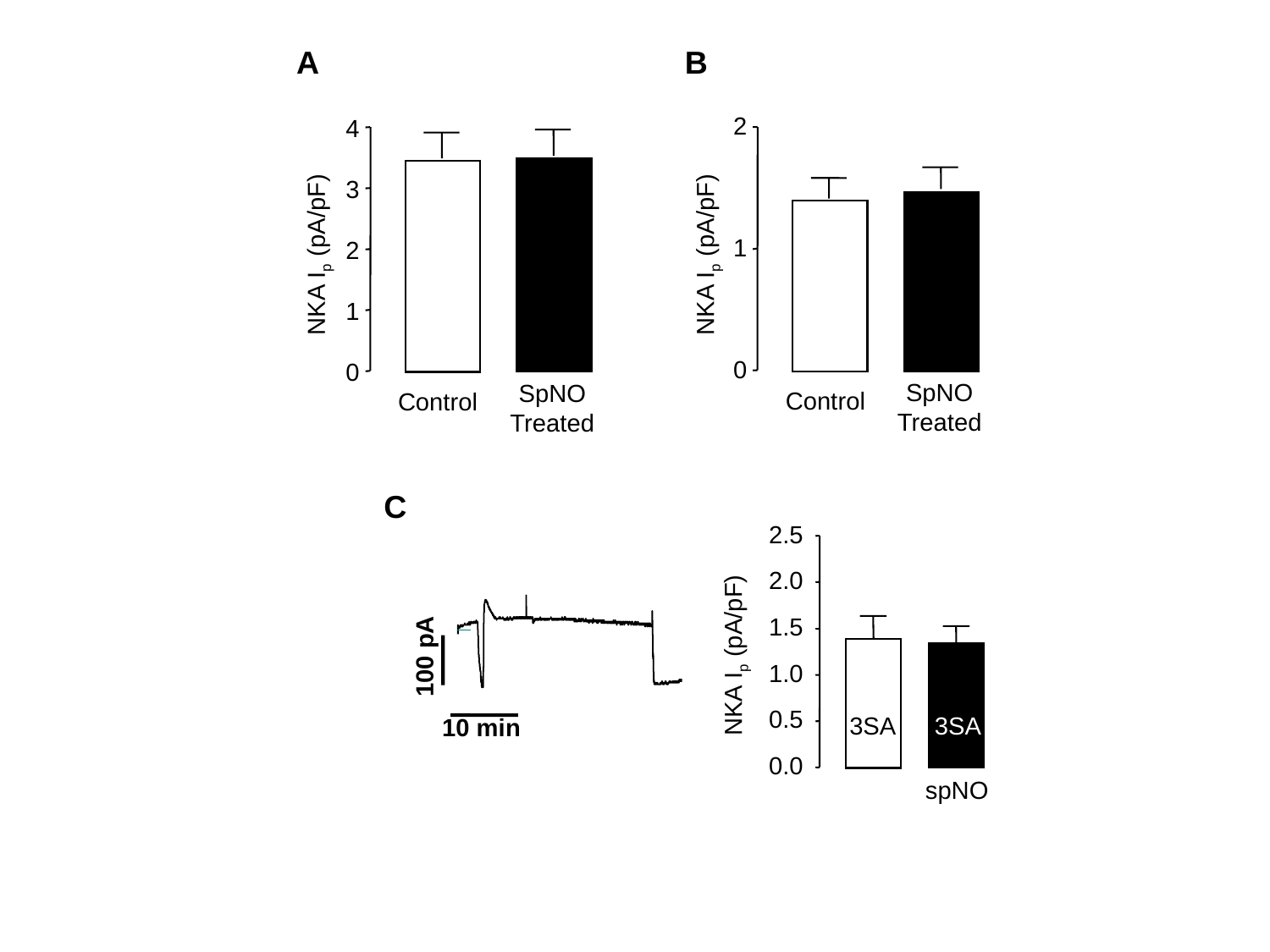

A
B
2
4
3
1
2
NKA Ip (pA/pF)
NKA Ip (pA/pF)
1
0
0
SpNO
Treated
SpNO
Treated
Control
Control
C
2.5
2.0
100 pA
10 min
NKA Ip (pA/pF)
1.5
1.0
0.5
3SA
3SA
0.0
spNO
