# Supplementary material for: Nitric oxide regulates cardiac intracellular Na+ and Ca2 + by modulating Na/K ATPase via PKCε and phospholemman-dependent mechanism
Source: J Mol Cell Cardiol. 2013 Aug;61:164–71. doi: 10.1016/j.yjmcc.2013.04.013 (PMC3981027; doi:10.1016/j.yjmcc.2013.04.013)
Supplement: Fig. S5 — NOS inhibition in field-stimulated rat myocytes results in elevation of Ca2 + transients and sarcomere length shortening and arrhythmias. Rat myocytes were field-stimulated from quiescence at 2 Hz in the presence of 1 mmol/L l-NAME. Raw traces of Ca2 + transients and sarcomere length shortening in the presence or absence of l-NAME (A). Changes in Ca2 + transients following field-stimulation, in the presence or absence of l-NAME (B). Examples of arrhythmias observed during field-stimulation in the presence of l-NAME (C). The data represent cells isolated from at least 3 individual animals and are expressed as mean ± sem (*P < 0.05 compared to non-treated control). [file mmc6.ppt]

## Slide 1
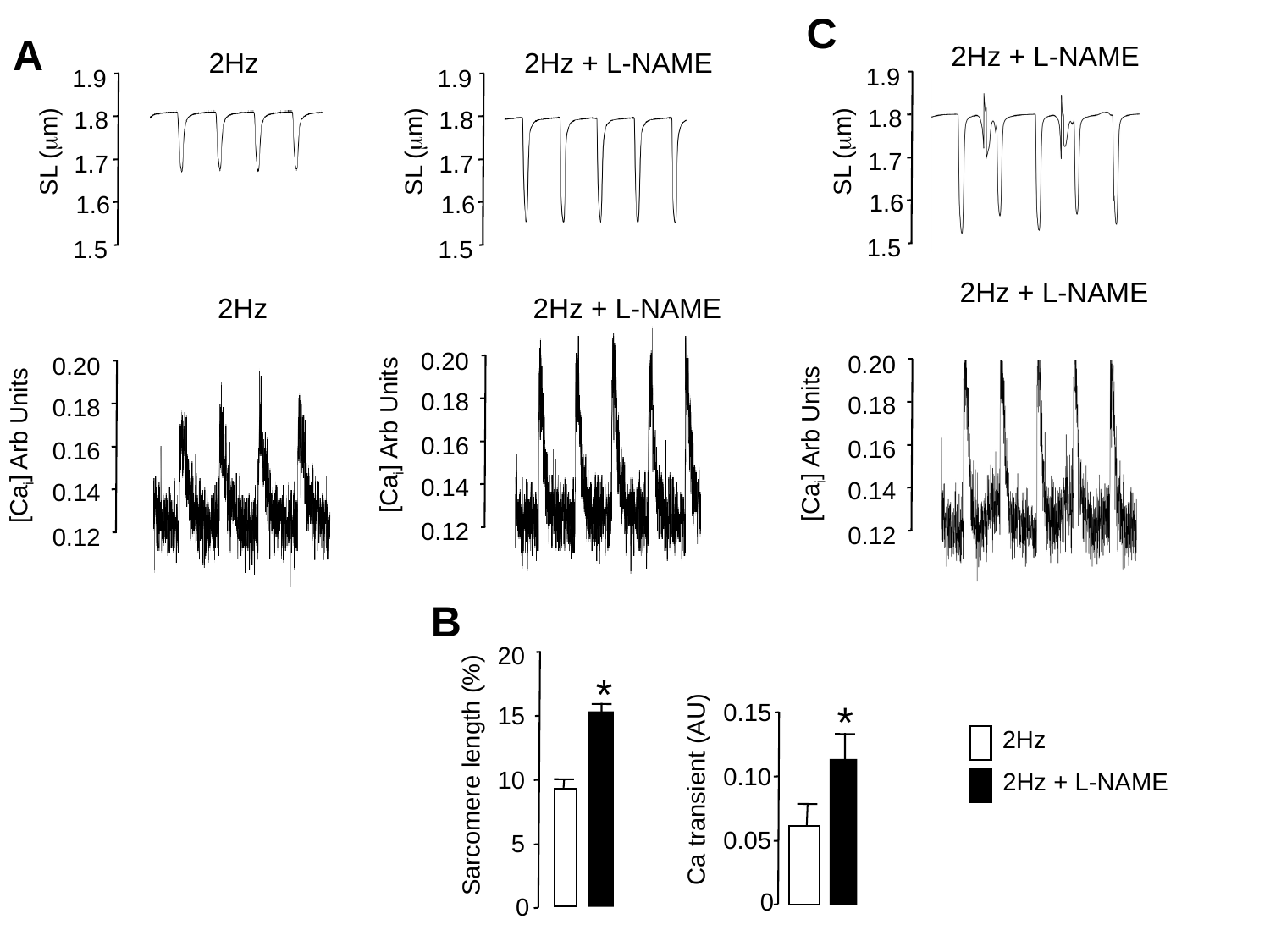

C
A
2Hz + L-NAME
2Hz
2Hz + L-NAME
1.9
1.8
1.7
1.6
1.5
1.9
1.9
1.8
1.8
SL (μm)
SL (μm)
SL (μm)
1.7
1.7
1.6
1.6
1.5
1.5
2Hz + L-NAME
2Hz
2Hz + L-NAME
0.20
0.18
0.16
0.14
0.12
0.20
0.18
0.16
0.14
0.12
0.20
0.18
0.16
0.14
0.12
[Cai] Arb Units
[Cai] Arb Units
[Cai] Arb Units
B
20
0.15
15
Sarcomere length (%)
0.10
10
Ca transient (AU)
0.05
5
0
0
*
*
2Hz
2Hz + L-NAME
