# Supplementary material for: Nitric oxide regulates cardiac intracellular Na+ and Ca2 + by modulating Na/K ATPase via PKCε and phospholemman-dependent mechanism
Source: J Mol Cell Cardiol. 2013 Aug;61:164–71. doi: 10.1016/j.yjmcc.2013.04.013 (PMC3981027; doi:10.1016/j.yjmcc.2013.04.013)
Supplement: Fig. S6 — Characterization of PLM3SA mice. Raw traces of the effects of forskolin on Ip in mouse myocytes isolated from PLMWT and PLM3SA animals, using perforated whole-cell patch clamp technique (A). Changes in Ip upon forskolin perfusion in mouse myocytes isolated from PLMWT and PLM3SA animals (B). Western blots showing changes in expression and phosphorylation of PLM (Ser-63, Ser-68, Ser-69), PLB (Ser-16, Thr-17), NKA α-1/2 and TnI (Ser-23/24) (C). Changes in expression of PLM and NKA α-1/2 (D). The data represent cells isolated from at least 5 individual animals and are expressed as mean ± sem (*P < 0.05 compared to control). [file mmc7.ppt]

## Slide 1
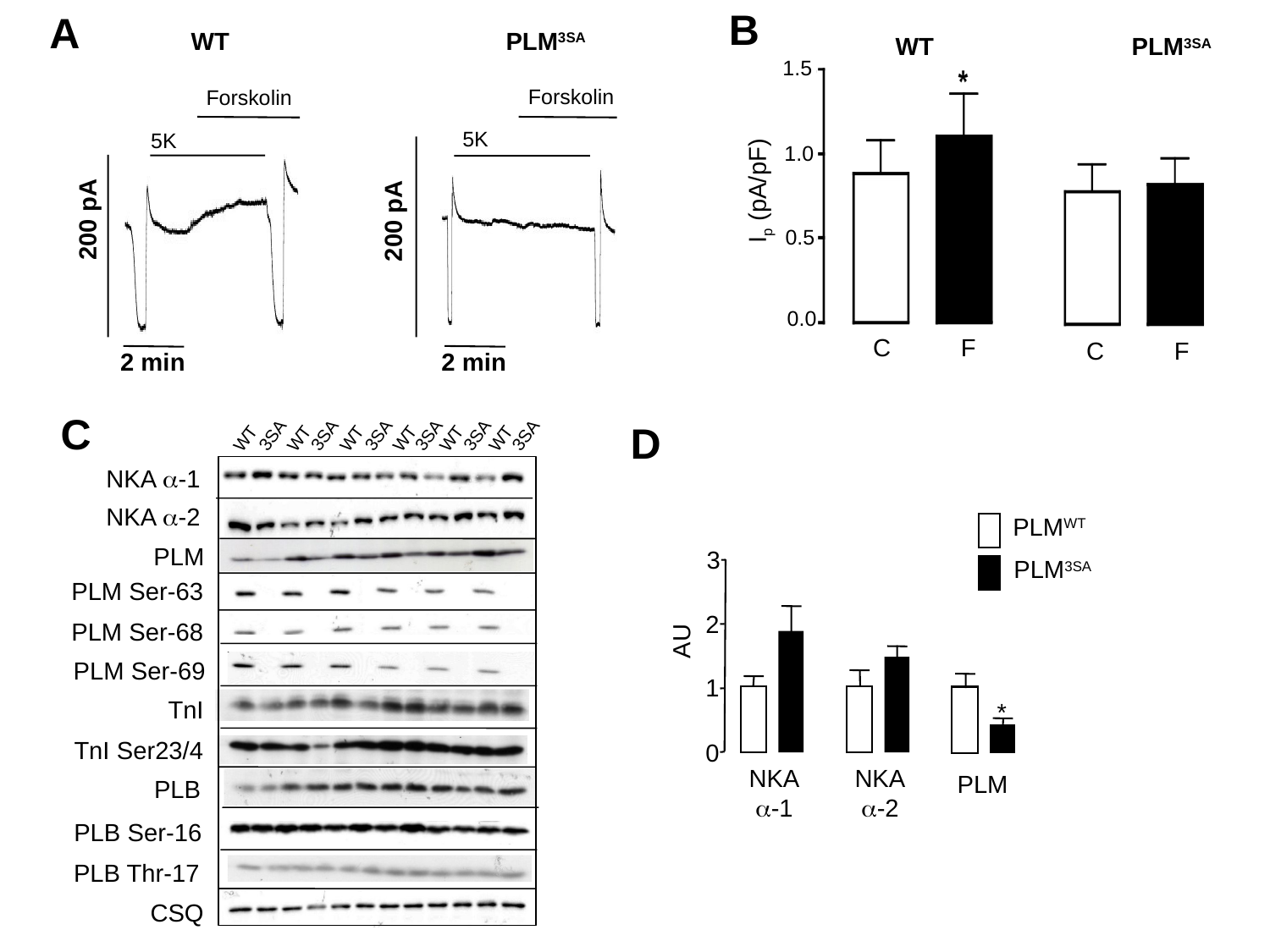

A
B
WT
PLM3SA
WT
PLM3SA
1.5
Forskolin
Forskolin
5K
5K
1.0
Ip (pA/pF)
200 pA
200 pA
0.5
0.0
C
F
C
F
2 min
2 min
C
D
3SA
3SA
3SA
3SA
3SA
3SA
WT
WT
WT
WT
WT
WT
NKA α-1
NKA α-2
PLMWT
PLM3SA
PLM
3
PLM Ser-63
2
PLM Ser-68
AU
PLM Ser-69
1
TnI
*
TnI Ser23/4
0
NKA
α-1
NKA
α-2
PLB
PLM
PLB Ser-16
PLB Thr-17
CSQ
